# Enhancing the Nutritional and Bioactive Properties of Bee Pollen: A Comprehensive Review of Processing Techniques

**DOI:** 10.3390/foods13213437

**Published:** 2024-10-28

**Authors:** María Alcalá-Orozco, Isabella Lobo-Farfan, Diego F. Tirado, Diana C. Mantilla-Escalante

**Affiliations:** 1 Universidad Nacional Abierta y a Distancia (UNAD), Sede Cartagena, Cartagena de Indias 130015, Colombia; diana.mantilla@unad.edu.co; 2 Cooperativa Multiactiva de Apicultores Orgánicos Montes de María (COOAPOMIEL), El Carmen de Bolívar 132050, Colombia; 3 Dirección Académica, Universidad Nacional de Colombia, Sede de La Paz, La Paz 202017, Colombia; ilobo@unal.edu.co; 4 Universidad del Sinú Elías Bechara Zainúm, Seccional Cartagena, Cartagena de Indias 1300001, Colombia

**Keywords:** superfood, exine, intine, sporopollenin, pollen-based product

## Abstract

Bee pollen is recognized as a superfood due to its high content of nutrients and bioactive compounds. However, its bioavailability is restricted by a degradation-resistant outer layer known as exine. Physical and biotechnological techniques have recently been developed to degrade this layer and improve pollen’s nutritional and functional profile. This review examines how processing methods such as fermentation, enzymatic hydrolysis, ultrasound, and drying affect pollen’s chemical profile, nutrient content, and bioactive compounds. The review also considers changes in exine structure and possible synergistic effects between these methods. In addition, the challenges associated with the commercialization of processed bee pollen are examined, including issues such as product standardization, stability during storage, and market acceptance. The objective was to provide an understanding of the efficacy of these techniques, their physicochemical conditions, and their effect on the nutritional value of the pollen. The work also analyzes whether pollen transformation is necessary to maximize its benefits and offers conclusions based on the analysis of available methods, helping to determine whether pollen transformation is a valid strategy for inclusion in functional foods and its impact on consumer health. Although the literature reports that pollen transformation influences its final quality, further studies are needed to demonstrate the need for pollen exine modification, which could lead to greater market availability of pollen-based products with functional properties.

## 1. Introduction

Beekeeping is a vital practice in agriculture and ecology, providing several products, including bee pollen [1], which is a complex food matrix composed of pollen grains that bees collect from flowers [2]. Bee pollen supplies nutrients for growth and development such as proteins, lipids, carbohydrates, minerals, vitamins, and phenolic compounds [3]. Such substances give pollen properties such as antioxidant, antibacterial, anti-inflammatory, immune system strengthening, anticancer, antibacterial, and immune system enhancement [2,4], which makes bee pollen recognized for high nutritional content and functional properties. However, bee pollen digestibility is limited, and the body does not entirely absorb its compounds [5]. This is due to the coating of the pollen by an outer layer known as exine, which is composed mainly of sporopollenin, an organic polymer resistant to the absorption processes of human metabolism [6,7]. This barrier limits the bioavailability of the pollen’s beneficial compounds, hindering their full health-promoting potential [8].

In recent years, research has been carried out on transformation processes in the exine structure to improve the absorption of compounds contained in pollen. These procedures can be performed through physical or biotechnological treatments [9]. This review aims to comprehensively address processing methods to optimize bee pollen’s nutritional and bioactive characteristics, focusing on fermentation, enzymatic hydrolysis, ultrasonography, and drying.

**Figure 1:**
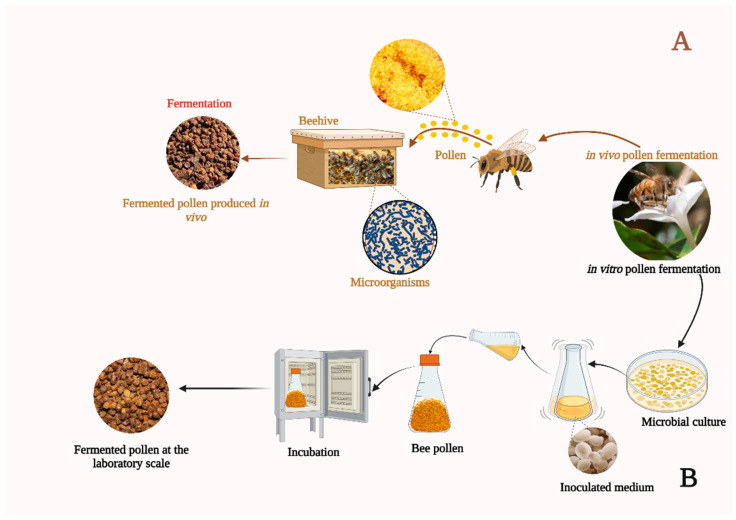
Graphical representation of the two ways of obtaining bee bread. (**A**) *in vivo* production, where bees collect and store pollen, which is then fermented with the help of microorganisms to form bee bread. (**B**) *in vitro* breeding, where a controlled fermentation with microorganisms, followed by incubation, is used to produce bee bread in the laboratory.

Fermentation is a microorganism-mediated biochemical process highlighted as an effective strategy to improve the bioavailability of intrinsic nutrients in bee pollen [10]. Through microbial metabolic activity, the synthesis or amplification of bioactive compounds such as amino acids, vitamins, and enzymes can be promoted, thus enriching the nutritional and functional value of pollen [9] (see Figure 1). Enzymatic hydrolysis, based on the action of specific enzymes, is an effective way to break down the complex polymers present in bee pollen (*e.g.*, carbohydrates and proteins) into more straightforward and more easily assimilated units (*e.g.*, monosaccharides and essential amino acids), improving the release and availability of nutrients and bioactive compounds, which could translate into health benefits for the consumer [11].

In the same way, ultrasonography is a non-thermal processing technique based on the phenomenon of acoustic cavitation [12]. It has been highlighted as an innovative method to improve the physicochemical characteristics of bee pollen. The application of high-frequency ultrasonic waves induces the rupture of pollen cell walls, facilitating the release of bioactive compounds and improving their bioavailability [13]. Finally, drying plays a crucial role in preserving the stability and quality of bee pollen during long-term storage. Conventional methods such as freeze-drying, convective drying, and spray-drying remove moisture from the pollen, minimizing the degradation of its nutritional and bioactive components [14,15]. This review provides a synthesis of how these techniques influence the functional properties of bee pollen, including digestibility, antioxidant activity, protein content, and phenolic compounds. A thorough search of several databases was conducted to ensure a comprehensive literature review, and reference lists of relevant articles were examined, ensuring the inclusion of relevant studies until July 2024.

## 2. Exine Composition and Function

**Figure 2:**
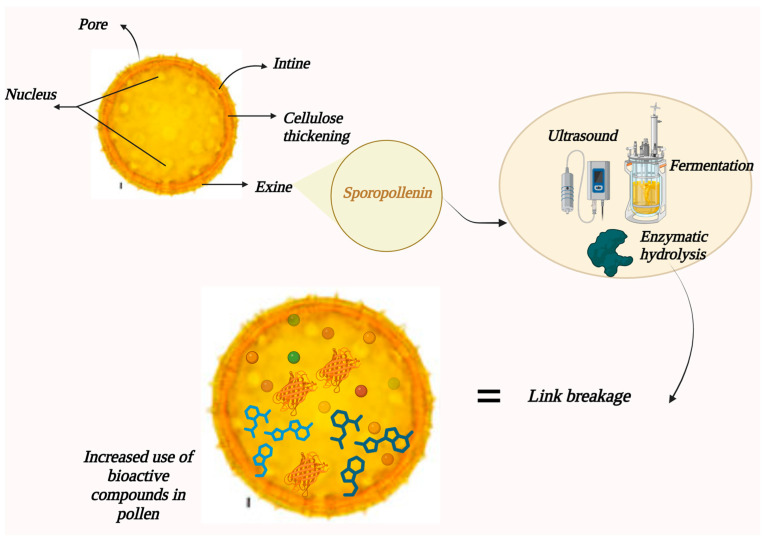
Graphical representation of how enzymatic hydrolysis, fermentation, and ultrasonication act synergistically on the pollen exine, weakening its structure to facilitate the release of bioactive compounds.

Pollen is an essential structure in the reproduction of flowering plants, acting as a carrier of male reproductive cells [16]. Its ability to withstand adverse environmental conditions is mainly due to the sporodermis, a complex membrane that protects the genetic material within the pollen grain [6]. The sporodermis comprises two main layers: the exine and the intine. The intine is an inner layer composed of cellulose and pectin; while the exine is the more rigid outer layer, rich in sporopollenin, which confers extraordinary chemical and biological resistance [16] (see Figure 2). Within its composition, sporopollenin includes long-chain fatty acids, phenylpropanoids, phenols, and trace amounts of carotenoids, all of which contribute to its hydrophobic properties, structural strength, and antioxidant capacity [17]. Consequently, the exine plays a crucial role in protecting pollen against environmental threats. The exine also exhibits considerable variability in its composition among different plant species [16], resulting in the diversity of patterns and ornamentations observed on the surface of pollen grains. This variability may influence the ability of pollen to interact with the environment and with the female reproductive structures of flowers [16,17].

Notwithstanding the importance of exine in protecting pollen, most studies on the denaturation of bioactive compounds in pollen have focused predominantly on intine [17]. The aforementioned needs to be clarified to understand the impact of exine modification on pollen’s bioactive properties. In this context, it is essential to investigate the effects of combined denaturation methods such as enzymatic hydrolysis, fermentation, and ultrasonication on the exine structure and how these techniques may influence the release and extraction of bioactive compounds from pollen.

## 3. Applied Techniques in Exine Denaturation

Nowadays, advanced techniques for pollen processing have emerged [18,19]. These techniques range from traditional methods such as fermentation and drying, to modern innovations such as enzymatic hydrolysis and ultrasound treatment [10,11,12,15]. All these technologies aim to denature the pollen exine [17], increasing the bioavailability of nutrients and bioactive compounds. Thus, this section was devoted to exploring how these techniques revolutionized the field, facilitating access to nutrients, and expanding pollen’s nutritional and medicinal applications.

### 3.1. Bee Pollen Fermentation

Fermentation is a biological process in which microorganisms, such as bacteria and fungi, break down organic compounds [20]. In the context of exine modification, fermentation can induce alterations in the composition and structure of this outer layer by partially degrading the sporopollenin and other organic components [21]. During fermentation, microorganisms secrete enzymes and metabolites that interact with the exine, breaking chemical bonds and modifying its structure [9,22]. Such a process contributes to the weakening of the exine and facilitates the release of previously inaccessible bioactive compounds, thus improving their availability for downstream application [2,9]. Moreover, fermentation greatly increases the total content of phenolic compounds in pollen due to their release after degradation of the pollen cell wall, thus enhancing its antioxidant and radical scavenging activity [23]. Essential phenolic compounds in fermented pollen include naringenin, quercetin, luteolin, and rutin, though their amounts vary with pollen origin and fermentation method [23,24].

Pollen fermentation is complex and includes both natural and artificial methods, each affecting the physicochemical and biological properties of the products [25]. Natural fermentation is an intrinsic process that occurs spontaneously under environmentally suitable conditions, driven by a consortium of indigenous microorganisms inhabiting the pollen matrix [26]; these include bacteria such as *Pseudomonas* spp. and *Lactobacilli* spp., as well as yeast strains like *Saccharomyces* spp. These microorganisms catalyze biochemical transformations within the pollen substrate, yielding structural and compositional changes [27]. Natural fermentation starts soon after pollen harvesting, with increased activity in beehives where interactions among pollen, bee honey, and bee saliva stimulate microbial activity and enzymatic degradation, leading to the formation of bee bread (see Figure 1A) [28]. Also, this fermentation enhances the biological activities of bee pollen, such as its antimicrobial and antioxidant properties through contributions from both the natural microflora and added bacteria [29]. Despite the aforementioned benefits of natural fermentation, the difficult scalability of this mechanism is a practical and economic challenge that requires alternative approaches [9]. Finally, Anderson *et al.* [30] found that there is no bacterial activity on stored pollen.

On the one hand, artificial fermentation can be achieved by adding pollen to a solution and subjecting it to controlled fermentation at different temperatures, allowing large-scale pollen production with specific properties [9]. Likewise, bee bread made with starter cultures (see Figure 1B) exhibits better microbial stability, which improves food safety and extends shelf life [9]. Also, starter cultures reduce fermentation time and produce granules with a texture and consistency like natural bee bread [9]. This enhancement benefits the product’s appearance, digestibility, and nutritional value, as reflected in improved amino acid profiles [24]. Finally, both natural and artificial fermentation can be contaminated by different microorganisms, including bacteria and molds [21]. In such cases, sterilization methods ensure safety and quality by eliminating unwanted microorganisms while preserving essential components and sensory characteristics [31].

Researchers are now focusing on simulating pollen fermentation in controlled laboratory settings [21], which involves inoculating pollen grains with specific microorganisms to replicate the biochemical changes seen in natural fermentation. By carefully controlling fermentation conditions, researchers aim to enhance the nutritional and functional qualities of the resulting product, ensuring that the artificial bee bread mirrors the characteristics of its natural counterpart [25]. Additionally, solid-state lactic acid fermentation has successfully improved bee pollen’s nutritional and antioxidant properties, enhancing bioactive compound release and potentially replicating natural fermentation in controlled environments [23].

### 3.2. Enzymatic Hydrolysis of Bee Pollen

The enzymatic hydrolysis of pollen is a biochemical process that breaks down complex polysaccharides, proteins, and other macromolecules into more minor, more absorbable compounds [32]. Enzymatic hydrolysis applied to bee pollen employs specific enzymes to catalyze the breaking of covalent chemical bonds within the sporopollenin [32]. This weakening not only facilitates the partial degradation of the exine but also increases the permeability of the outer layer, allowing other denaturing agents to penetrate deeper into the pollen grain [11].

Unlike fermentation, which uses microbial activity to drive biochemical changes, enzymatic hydrolysis employs specific proteins selected for their efficiency and precision under stringent quality standards [33]. Additionally, precise control in enzymatic hydrolysis provides specific product characteristics, distinguishing it from fermentation and enhancing product quality [11]. This distinction impacts several fields, including food and biotechnology, where both methods produce products with distinct properties and applications [22]. Finally, the selection of proteins for the enzymatic hydrolysis of pollen is governed by critical criteria in generating bioactive peptides [11].

In biological hydrolysis, enzyme selection is crucial for determining the composition and properties of the pollen product [32]. Several enzymes, including proteases, carbohydrases, and lipases are chosen to target specific components within the pollen matrix, breaking down complex macromolecules into more accessible forms [32,34]. Proteases catalyze the hydrolysis of peptide bonds, releasing essential amino acids and peptides that enhance the nutritional profile of the hydrolyzed product [33]. For example, Pasarin and Rovinaru [1] compared hydrolysates from bee pollen using two commercial enzymes: Alcalase 2.4 L and Protamex. These authors found Alcalase 2.4 L had a slightly higher proteolytic activity (10.4%) than Protamex (9.3%), improving the product’s commercial and nutritional value [34]. Similarly, Zuluaga-Domínguez *et al.* [11] observed a 13–18% increase in protein content in pollen after treatment with proteases. Likewise, carbohydrases break down complex carbohydrates in bee pollen into simpler sugars, improving their energy utilization and functional properties [5]. In this regard, Damulienė *et al.* [32] found that bee pollen treated with cellulase, Viscozyme^®^ L, and Claradiastase had 1.5 to 2.5 times more flavonoids than those treated with lipase, protease, and amyloglucosidase [32].

Finally, enzyme selection, such as bromelain, proline, and lipase, is based on substrate specificity, activity, and efficiency [35]. Tailoring the enzyme mix to the pollen composition allows for precise control over the hydrolysis process, resulting in pollen with targeted nutritional, functional, and sensory properties [32].

### 3.3. Ultrasound

Ultrasonication is a technique that uses high-frequency sound waves to generate cavitation and microagitation in liquid media [13]. When applied to the pollen exine, ultrasonication causes the formation of cavitation bubbles that implode, generating intense mechanical forces that disintegrate cellular structures and protective layers [36]. This process physically fragments the exine and increases the contact surface for other denaturing processes, such as enzymatic hydrolysis and fermentation [37]. Additionally, by promoting the physical disruption of the exine, ultrasonication facilitates the release of bioactive compounds trapped inside, making them more accessible for extraction and subsequent use in various industrial and therapeutic applications [38].

Ultrasound technology in bee pollen processing promises to improve product quality and functionality, drawing significant attention for its non-invasive nature and efficient process enhancement potential [39,40]. Thus, ultrasound technology has been recognized as a valuable wall-disruption technique for promoting nutrient release and enhancing pollen’s biological effects [41,42,43,44,45].

## 4. Optimal Pretreatment for Enhanced Bee Pollen Processing

One of the biggest challenges in commercializing bee pollen is its susceptibility to biocontamination due to its high nutritional content and the poor aseptic conditions of beehives and apiaries [46]. Thus, pretreatments must be applied to ensure appropriate conditions for biological activity and product quality [22,47,48]. Maintaining pollen viability is critical [49]; so, pretreatment must preserve the viability of pollen grains, ensuring that they stay alive and are able to germinate [50].

The bee pollen moisture content must be below 10% to avoid its degradation [51]. To achieve this, drying is usually used as pretreatment [47]. However, drying is an energy-intensive operation, accounting for about 20% of the total energy consumed in the food industry, in which more than 85% of the dryers are convective, with hot air as the main heat transfer medium [52]. In addition to energy efficiency, the retention of pollen quality is paramount; so, precise temperature control is crucial to keep pollen’s structural integrity and bioactive properties, avoiding degradation, and ensuring quality [51].

Freeze-drying is one of the most effective methods for preserving bee pollen, as it involves sublimating water from the frozen state directly into vapor without passing through the liquid phase [53]. This gentle method preserves heat-sensitive bioactive compounds, including phenolics, flavonoids, and amino acids, ensuring the highest possible retention of nutritional and functional properties. The process involves freezing the bee pollen at very low temperatures, then reducing the pressure and adding heat to allow the frozen water to sublimate [18,51]. As a result, freeze-dried bee pollen retains its original structure, color, flavor, and bioactive compounds better than other drying methods [53]. Although effective, freeze-drying requires specialized equipment, such as freeze dryers, which can limit availability and extend processing times, affecting efficiency and productivity [53]. Thus, this method is expensive and time-consuming, making it less suitable for large-scale industrial applications [18].

Meanwhile, convective drying or air-drying, is a standard method where heated air is passed over the bee pollen to evaporate moisture [54]. The efficiency of convective drying depends on factors such as temperature, airflow rate, and humidity [40,54]. While this method is more cost-effective and faster than freeze-drying, it may lead to the degradation of heat-sensitive compounds, loss of volatile components, and changes in color and texture [54,55]; so, the drying temperature must be carefully chosen and controlled to minimize the loss of nutrients and bioactive compounds [51].

Alternatively, spray-drying is widely used in industrial applications to turn liquid bee pollen extracts into a stable powder form [56,57]. The process involves atomizing the liquid extract into a hot air chamber, where rapid moisture evaporation occurs. Spray-drying offers a rapid and continuous method for producing dry pollen with good solubility and ease of handling [58]. However, the high temperatures in spray-drying may lead to the degradation of heat-sensitive components, such as specific vitamins and phenolic compounds [59]. Thus, lower inlet air temperatures or protective carriers like maltodextrin may be used to mitigate these effects [60].

Meanwhile, solar-drying is crucial for altering the external structure of pollen grains, enhancing nutrient availability, and reducing microbial contamination [61]. On the other hand, microwave-drying uses electromagnetic waves for rapid and energy-efficient heating and dehydration, preserving bioactive compounds with minimal heat exposure [62]. However, the careful control of microwave power and moisture content is necessary to avoid overheating and ensure product quality [62].

Finally, vacuum-drying reduces the pressure around the bee pollen, allowing water to evaporate at lower temperatures [63]. This method is beneficial for preserving heat-sensitive compounds, like freeze-drying, but is faster, more cost-effective, and therefore scalable [53]. The vacuum-drying process results in a product with a lower risk of oxidation and nutrient degradation, making it suitable for preserving the quality of bee pollen [40]. However, like other drying methods, the careful control of processing conditions is required to maintain the bioactive compounds’ integrity [57].

Achieving optimal drying outcomes involves considering product characteristics and processing scale [54]. Mechanical fragmentation, such as milling or grinding, increases pollen’s surface area for biological processes, enhancing hydrolysis or fermentation efficiency [62]. Grinding improves nutrient and bioactive compound availability, increasing the digestibility and assimilation of proteins, lipids, vitamins, and polyphenols [45]. This fragmentation also aids enzymatic actions and nutrient release during hydrolysis or fermentation [64]. After fragmentation, sieving ensures uniform particle size distribution, consistency in morphology, and the removal of coarse debris and impurities [65]. The particle size of pollen significantly affects its susceptibility to ultrasound treatment, with smaller sizes improving ultrasound transmission and penetration, enhancing effectiveness [66]. Optimal sizes typically range from 100 µm to 500 µm, and techniques such as laser diffraction or sieving ensure consistency [39].

Keeping the right temperature conditions during pretreatment prevents the thermal degradation of sensitive components like enzymes, microorganisms, and phytochemicals [40,67]. Cooling methods, such as cryogenic grinding or controlled temperature environments, help to prevent heat-induced damage and preserve quality [54]. Additionally, adjusting moisture levels is essential to prevent spoilage and extend shelf life, with low moisture content inhibiting microbial growth [7,53,68].

Moreover, achieving a uniform particle size and moisture content is essential for optimal ultrasound penetration and absorption [69]. Excessive moisture can hinder ultrasound energy transfer, potentially leading to ineffective treatment or product degradation [36,70]. Conversely, overly dry pollen may not absorb ultrasound energy effectively, reducing treatment efficacy. The ideal moisture content typically ranges from 5% to 10%, though this can vary based on treatment goals [13,45]. After pretreatment, samples are subjected to strict quality control to ensure purity and consistency [71].

Response surface methodology (RSM) has been used to evaluate pretreatment parameters and identify optimal conditions [43,60]. Likewise, factorial design experiments have explored the effects of milling time, temperature (ambient or controlled), and sonication speed on particle size and moisture content [72]. Both drying and particle size reduction are energy intensive, so they are operations that must be optimized for the commercialization of the process.

Finally, pollen is often sterilized using methods such as pasteurization and irradiation to eliminate microbial contamination [73]. Sterilization guarantees microbiological safety and prevents unwanted fermentation [31]. Monitoring particle size distribution, moisture content, and temperature parameters helps detect deviations early and enables corrective actions to maintain product quality. Implementing quality assurance protocols such as good manufacturing practices (GMP) and hazard analysis and critical control points (HACCP) minimizes contamination risks and ensures product safety [74].

## 5. Conditions of Bee Pollen Processing Techniques

Understanding the conditions under which processing techniques occur is crucial for optimizing product quality. This section provides comprehensive insights into how precise control over these processes contributes to the desired outcomes.

The fermentation of bee pollen mainly involves solid-state fermentation (SSF) and liquid-state fermentation (LSF), each with distinct conditions and microbial interactions aimed at enhancing the product’s final nutritional and functional properties [75]. SSF typically occurs within beehives, where pollen is stored in cells treated with salivary secretions and 10-hydroxy-2-decenoic acid, then covered with honey to create an anaerobic environment at about 35 °C. This process turns pollen into bee bread through bacterial and yeast action [76].

Despite its advantages, SSF faces challenges such as heat accumulation, bacterial contamination, microorganisms that can grow at low water activity levels, and difficulties in moisture control, microbial growth determination, and product purification [26]. On the other hand, LSF cultivates microorganisms in liquid substrates like broths, allowing better nutrient diffusion and faster microbial growth [25]. This method is used to produce probiotic-rich bee pollen supplements or functional beverages. Uțoiu *et al.* [56] found that Kombucha fermentation improved pollen’s bioactive compounds, such as polyphenols and soluble silicon, and showed a moderate antitumoral effect on Caco-2 cells. Both solid-state (SSF) and liquid-state (LSF) fermentations are influenced by factors like temperature, pH, and oxygen availability, which impact microbial interactions and product quality [25]. These aspects require careful consideration in fermentation process design and optimization.

Parameters such as pH, temperature, enzyme concentration, and reaction time should be carefully controlled during pollen hydrolysis to achieve desired outcomes, maximizing enzyme activity and substrate conversion, while minimizing side reactions [77]. Protease-mediated hydrolysis, for instance, typically occurs under acidic-neutral conditions (pH 4.0–8.0) from 40 °C to 60 °C, utilizing enzymes such as papain [48]. Likewise, carbohydrase-mediated hydrolysis requires neutral to slightly acidic conditions (pH 4.0–6.8) between 30 °C to 50 °C, employing enzymes like α-amylase [42]. Meanwhile, lipase-mediated hydrolysis operates optimally at a neutral to alkaline pH (pH 7.0–8.0) from 30 °C to 50 °C, with enzymes such as lipase being utilized [32].

Parameters involved in applying ultrasound technology to bee pollen also require thorough attention to achieve the desired outcomes. These parameters include frequency, power density, treatment duration, and temperature control. Adapting these factors to specific objectives, such as enhancing nutritional content, reducing microbial load, or improving digestibility, demands careful consideration and systematic optimization [32,35].

Ultrasound frequency affects penetration depth and biological effects. Higher frequencies (*e.g.*, 80 kHz) are ideal for surface treatments, pollen wall disintegration, and microbial inactivation [39,42,69]. Lower frequencies (*e.g.*, 20 kHz–40 kHz) offer deeper penetration, making them suitable for structural modifications or extractions [38,53]. The optimal frequency depends on empirical studies and varies by application and equipment.

Power density measures ultrasound energy per unit area (*i.e.*, W/cm^2^). Higher power densities speed up treatment, but risk thermal damage and alterations in pollen properties, while lower densities reduce these risks but may require longer treatment times. Optimizing power density involves balancing efficiency with product quality [37]. Treatment duration depends on the extent of modification, sensitivity of pollen components, and ultrasound parameters. Shorter durations are suitable for surface disinfection or particle size reduction, while longer durations are needed for structural changes or bioactive compound extraction [44].

Maintaining an appropriate temperature during ultrasound treatment is essential to avoid thermal damage and preserve bee pollen’s nutritional and functional integrity [13]. Optimal temperatures generally range from 20 °C to 48 °C, though specific needs depend on pollen sensitivity and ultrasound parameters [39]. Excessive heat can cause protein denaturation, enzymatic degradation, and loss of bioactive compounds [51]. Water baths, cooling jackets, or ultrasound probes with temperature regulation systems help control heat and ensure consistent treatment [40].

## 6. Quality Assurance and Product Refinement

Following fermentation, post-processing steps refine the pollen product to meet quality and safety standards. These techniques include filtration, concentration, drying, grinding, packaging, and rigorous quality control measures such as microbial testing, chemical analysis, and sensory evaluation [25]. These steps ensure high-quality fermented pollen suitable for various applications and consumer preferences. Similarly, after enzymatic hydrolysis and ultrasound treatments, post-treatment steps are crucial to refine, stabilize, and enhance the product [22]. This phase involves filtration to remove insoluble residues, impurities, and particulate matter, improving clarity and purity [12]. Techniques such as evaporation or membrane filtration concentrate bioactive compounds, increasing potency [78]. Quality control also evaluates sensory properties such as taste, aroma, texture, and overall palatability to ensure consumer acceptance [79].

Proper packaging protects bee pollen products from contamination, oxidation, and moisture ingress during storage and transportation. Food-grade polyethylene bags, glass jars, or vacuum-sealed pouches are commonly used, selected for their barrier properties and compatibility with the product [80]. Packaging may also include oxygen scavengers, desiccants, or moisture barriers to enhance stability and shelf life [81]. In addition to suitable packaging, appropriate storage conditions are essential. Freezing helps preserve bee pollen’s sensory and nutritional qualities, including sugars, lipids, phenols, and flavonoids [82]. Controlled environments (*e.g.*, cool, dark, and dry) are essential to minimize oxidative reactions, microbial growth, and moisture uptake, thereby maintaining product integrity [83]. Monitoring and controlling temperature and humidity and using ventilation and pest control measures further ensure the preservation of bioactive compounds and sensory attributes [56].

Finally, quality assessment protocols are crucial for evaluating treatment efficacy, product stability, and adherence to regulatory standards. Microscopy, spectroscopy, chromatography, and microbiological assays are used to analyze key parameters like particle size, nutritional composition, microbial load, and sensory attributes. Regular monitoring throughout the post-treatment process helps detect deviations from quality standards early and allows for corrective actions, ensuring product integrity and safety [81,82].

## 7. Effects of Processing Methods on Bee Pollen Functional Properties

Many studies have explored the impact of fermentation on bee pollen, investigating its functional properties and biological activities, as can be seen in Table 1. Fermentation with *Saccharomyces cerevisiae* and lactic acid bacteria led to a significant increase in total phenolics, and antioxidant activity compared to fresh bee pollen [10]. Similarly, fermentation with selected strains of *Lactobacillus kunkeei* and *Hanseniaspora uvarum* resulted in the enhanced nutritional value and microbial stability of bee-collected pollen, with notable increases in peptides and amino acids [84]. Another study demonstrated that fermentation with *Lactococcus lactis* and *Lactobacillus rhamnosus* significantly improved bee pollen’s antibacterial, antifungal, and antioxidant activities [29]. Fermentation with *S. cerevisiae* also increased the nutritional value and bioactive composition of bee pollen, along with its antioxidant and anti-inflammatory activities [7].

Different fermentation methods have been shown to enhance bee pollen’s properties. Using starters like *Lactobacillus acidophilus* and *Lacticaseibacillus rhamnosus* improved lactic acid production and antioxidant activity [23,68]. Kombucha fermentation increased bioactive compounds and antitumor effects [56]. Yeast fermentation helped prevent metabolic syndrome by modulating gut microbiota [85]. Ultrasound-assisted fermentation with *L. rhamnosus* and *S. cerevisiae* also boosted bioactive composition and antioxidant activity [45]. These findings highlight fermentation’s potential to improve bee pollen’s functional and biological benefits in functional foods and nutraceuticals [4].

The enzymatic hydrolysis of bee pollen has been extensively studied to improve its bioactive composition and nutritional value. Different enzymes, including Alcalase, Protamex, Viscozyme, Claradiastase, lipase, amyloglucosidase, cellulase, pectinase, papain, and pepsin have been used to break down pollen proteins and other constituents [71]. Research indicated that protease hydrolysis can significantly boost protein content by up to 18%, phenolics by up to 106%, flavonoids by up to 96%, and antioxidant activity by up to 68% [11]. Enzymes like Viscozyme^®^ L, Claradiastase, and lipase also enhanced the phenolic and flavonoid content, radical scavenging, and antibacterial activities [32]. Alcalase and Protamex improved nutritional value and proteolytic capacity [1], while gastrointestinal enzymes such as pepsin and trypsin produced bioactive peptides with high angiotensin-converting enzyme (ACE)-inhibitory and antioxidant activities [34].

The application of enzymes such as bromelain, aminopeptidase, and proline aminopeptidase has led to hydrolysates with an increased hydrolysis degree, phenolic content, protein content, and antiradical scavenging activity [35]. Enzyme-treated bee pollen has also shown potential in reducing allergenicity, evidenced by decreased scratching frequency in mice, improved histopathological outcomes, lowered serum IgE levels, and modulated metabolic pathways and gut microbiota [33].

Additionally, treatments like ultrasonication, freeze-thawing, and high-shear techniques have been explored to improve the physicochemical properties of bee pollen [86]. These methods help disrupt the pollen wall, facilitating nutrient and bioactive compound release. Combining enzymatic hydrolysis with ultrasonication or freeze-thawing has shown to increase protein yield and enhance functional properties, as demonstrated by Xue and Li [48], who found that these combined treatments improved protein yield and functionality through changes in protein structure.

Studies have demonstrated the efficacy of ultrasonication and high-shear techniques in breaking down pollen walls, leading to increased levels of amino acids, fatty acids, proteins, and other nutrients [86]. Ultrasonic extraction has been optimized to boost bee pollen’s total phenolic content and antioxidant activity [43]. Additionally, green extraction methods like microwave- and ultrasound-assisted extraction have shown enhanced yields of bioactive compounds, with microwave-assisted extraction achieving the highest phenolic and flavonoid content [2]. Also, techniques for wall disruption such as ultrasonication and high-shear techniques have improved the digestibility and bioavailability of bee pollen’s bioactive compounds. For example, Protamex hydrolysis combined with ultrasonication has increased the specific surface area and reduced particle sizes. Studies on wall-disruption methods using Protamex hydrolysis and ultrasonication have shown an increased specific surface area and smaller particle sizes than untreated samples. These approaches enhance bee pollen’s nutritional value, antioxidant activity, and bioactive composition, underscoring its potential as a functional food ingredient [39].

Different drying techniques have also been explored to improve bee pollen’s nutritional content and antioxidant properties. Fresh bee pollen samples were subjected to microwave-drying, microwave-assisted vacuum-drying, hot-air-drying, vacuum-drying, and freeze-drying, as well as methods to reduce moisture content. Microwave-assisted vacuum-drying and treatments at lower power levels exhibited a better retention of nutrients like vitamins C and E than hot-air-drying and freeze-drying. Likewise, microwave-drying resulted in higher antioxidant activity than hot-air-drying, irrespective of the pressure or power level applied [15].

Pulsed vacuum-drying (PVD) and freeze-drying were compared for their effects on the amino acid release, α-dicarbonyls content, and volatile compounds in bee pollen. Freeze-drying notably increased the release of essential amino acids, while PVD mitigated quality deterioration in raw bee pollen [51]. Oven-drying and lyophilization were evaluated for their impact on the biochemical profile of bee pollen. Lyophilized samples exhibited higher concentrations of flavonoids and phenolic compounds and more potent antimicrobial and antioxidant activities than oven-dried samples [87].

Studies on solar and microwave-drying methods have revealed that lower power levels in microwave-drying better preserve bee pollen quality than higher levels [88]. Comparisons between hot-air and vacuum-drying showed that vacuum-drying at lower temperatures kept higher total phenolic and flavonoid contents with less color change [62,88]. These findings highlight that drying methods differently impact bee pollen’s nutritional, bioactive, and antioxidant properties. Thus, choosing the proper drying technique depends on the desired preservation of bioactive compounds. Finally, further research is necessary to optimize drying parameters and understand the mechanisms affecting dried bee pollen quality.

**Table 1:** Functional properties identified in processed pollen.

Identified Functional Property	Technique	Biological Agent	Assay Conditions	Main Observations	Reference
Antioxidant effect	Fermentation	*S. cerevisiae*, *L. plantarum*, commercial culture Choozit^®^	Six different fermentation tests were conducted. Two tests involved inoculating yeasts using ATCC and commercial cultures, respectively. Another two tests involved inoculating lactic acid bacteria from ATCC strains and commercial cultures. The final two tests utilized consortia of yeast and lactic acid bacteria, one from ATCC strains and the other from commercial cultures. For each preparation, 200 g of substrate was prepared using a 1:1 ratio of bee pollen and water, with the inoculum incubated at 37 °C for 72 h.	Compared to fresh bee pollen, the fermented products exhibited a 31% increase in total phenolic content and a 39% enhancement in antioxidant activity.	[10]
Increased nutritional value for humans and microbial stability	Fermentation	Mixed inoculum of selected *L. kunkeei* strains and *H. uvarum* AN8Y27B	The bee pollen was fermented with a mixed inoculum consisting of *L. kunkeei* strains PF12, PL13, and PF15, along with *H. uvarum*, in sealed tubes at 30 °C for 216 h. Sterile water was added to achieve a final water content of 40% *w*/*w*.	The concentration of peptides in raw bee pollen was (14 ± 2) mg/kg dry weight, which increased to (51 ± 2) mg/kg dry weight following fermentation. Additionally, the total free amino acids significantly increased during fermentation. The microbial stability of pollen during storage at room temperature (25 °C) for 50 days showed a decreasing trend in lactic acid bacteria, with a significantly slower reduction observed in started pollen compared to the raw material. Yeasts exhibited higher cell densities in raw pollen, while molds were present for up to 20 days, with the highest cell density observed in the raw and unstarted material.	[84]
Antibacterial, antifungal, and antioxidant activities	Fermentation	*L. lactis* and *L. rhamnosus*	The inoculum was cultured statically in MRS broth containing Tween 80 at 37 °C until the cell density was measured spectrophotometrically at 600 nm.	After fermentation, total phenolic content, total flavonoid content, and radical scavenging activity increased by 1.3 to 2.4 times. The antibacterial activity against *Micrococcus luteus*, *Staphylococcus aureus*, and *Escherichia coli* increased by 1.1 to 16.9 times, while antifungal activity against *Penicillium roqueforti* rose by 2.0 to 5.5 times. The natural microflora of the bee pollen, in conjunction with the added bacteria, contributed to the fermentation process. Consequently, pasteurized bee pollen exhibited significantly lower antimicrobial and antioxidant activities compared to fermented natural bee pollen.	[29]
Improved nutritional value	Fermentation	*S. cerevisiae*	The activation of *S. cerevisiae* in sterile water at 37 °C for 1 h, followed by its transfer to sterilized bee pollen at a carefully measured ratio of yeast to bee pollen (1:50 *w*/*w*) and bee pollen to sterile water (1:20 g/mL), and subsequent culture at 160 rpm in a 1 L shaking flask at 28 °C for 8 days, demonstrated the meticulous nature of the experiment. Ultra-performance liquid chromatography–electrospray ionization–mass spectrometry (UPLC-ESI-MS) was employed for a widely targeted metabolomics analysis to compare the chemical composition of unfermented bee pollen and fermented bee pollen.	Fermentation significantly increased the levels of primary metabolites, including 74 amino acids and their derivatives, 42 polyunsaturated fatty acids, and 66 organic acids. Additionally, it elevated the concentrations of certain secondary metabolites, such as 38 phenolic acids, 80 flavone aglycones, and 22 phenol amides.	[7]
Antioxidant and anti-inflammatory activities	Fermentation	*S. cerevisiae*	*S. cerevisiae* was activated in sterile water at 37 °C for 1 h, then transferred to sterilized bee pollen and cultured at 160 rpm in a 1 L shaking flask at 28 °C for 8 days.	Fermentation significantly enhanced antioxidant activity (by more than 2.3-fold) and anti-inflammatory activity (by more than 1.4-fold) of the bee pollen and increased the contents of total phenolics and flavonoids by 2.0-fold and 1.5-fold, respectively. Additionally, fifteen components, including three phenolamides, one flavonoid aglycone, seven fatty acids, three amino acids, and one ketone compound, were positively correlated with the antioxidant and anti-inflammatory activities of bee pollen.	[89]
Improved lactic acid production	Fermentation	Starter A (*Streptococcus thermophilus*, *Lactobacillus delbrueckii* ssp. *lactis*, and *L. delbrueckii* ssp. *bulgaricus*), starter B (*L. delbrueckii* ssp. *lactis*, *L. delbrueckii* ssp. *cremoris*, and *L. delbrueckii* ssp. biovar. *diacetylactis*), and starter C (*L. acidophilus* NCFM)	The substrate was prepared with a 10 min heat treatment, using a water ratio of 2:1. It was inoculated directly with 16 mg of starter culture/kg, placed in 6 L glass vessels without agitation, sealed, and incubated at (42.0 ± 0.5) °C for starters A and C, and at (37.0 ± 0.5) °C for starter B.	Among the starter cultures tested, the one consisting exclusively of probiotic *L. acidophilus* NCFM exhibited the highest performance in lactic acid production, achieving a concentration of 1.65% after 30 h.	[68]
Antioxidant activity	*in vitro* fermentation	*L. rhamnosus* GG (ATCC 53103)	A total of 10 g of each pollen sample were moistened with 2 mL of sterile distilled water for 2 h, then subjected to heating and cooling. A mixture of multifloral spring honey and water was added, followed by the addition of 800 µL of *L. rhamnosus* for bacterial fermentation and 800 µL of MRS broth with Tween 80 for spontaneous fermentation. Bacterial fermentation was carried out for 9 days, while spontaneous fermentation extended to 11 days, both at 37 °C.	The total phenolic content increased by 1.4 to 2.3 times, the total flavonoid content by 1.1 to 1.6 times, and the radical scavenging activity by 1.4 to 2.3 times. Naringenin, quercetin, luteolin, and rutin were the most abundant flavonoids found in all samples.	[23]
Antioxidant activity and increased nutritional value	*in vitro* fermentation	*L. lactis*	35 °C for the first 96 h and then 20 °C for next 72 h.	Proteins increased by 1.5%, while total sugars decreased by 32.6%. Lactic acid content rose by 1.4%, and total free amino acid content increased by 2.0%. Total polyphenol content decreased by 1.8%. All minerals showed an increase, and radical scavenging activity improved by 18.9% in the fermented pollen.	[20]
Antioxidant and antitumor activities, and increased nutritional value	*in vitro*fermentation	*Kombucha consortium*	Fermentation was conducted at 28 °C for 10 days using pollen and Kombucha vinegar, which was prepared by fermenting 5 g of green tea in 1 L of water at 28 °C for 20 days.	The content of bioactive compounds, including polyphenols, soluble silicon species, and short-chain fatty acids, was higher in the fermented pollen. The IC_50_ value for DPPH radical scavenging activity decreased progressively from 15.2 mg/mL on the first day of fermentation to 10.6 mg/mL on the 10th day. The product exhibited a moderate antitumoral effect on Caco-2 cells.	[56]
Antiviral activity against influenza a virus	Fermentation	*Apilactobacillus kunkeei*, *Zasphinctus siamensis*, *Bacillus licheniformis*, *Bacillus* spp. and *Bacillus subtilis*	Fermentation was performed at 33 °C for 28 days	IC_50_ values ranged from 0.022 mg/mL to 10.04 mg/mL, and Selectivity Index (SI) values ranged from 1.06 to 338.64. Artificially fermented pollen samples exhibited higher SI values compared to unfermented bee pollen, with the proteinaceous fractions showing the highest SI values.	[90]
Prevention of metabolic syndrome	Fermentation	*Yeast*	Fermentation was performed 37 °C for 48 h	Fermentation of pollen by yeast increased GST and CAT activities while reducing MDA levels in the liver through the restoration of the Nrf-2-Keap-1 pathway. Additionally, yeast-fermented pollen resulted in a decreased Firmicutes to Bacteroidetes ratio and an increased abundance of *Lactobacillus* and *Lactococcus*.	[85]
Improved bioactive composition and antioxidant activity	Ultrasound and fermentation	*L. rhamnosus* GG (ATCC 53103), *S. cerevisiae* strains	Bee pollen was finely ground using an MK-06M grinder. One batch of samples underwent ultrasound treatment (2 × 15 min, 700 W) in an ultrasonic bath to assess its effects on pollen structure and potential sterilizing properties. Ground bee pollen powder (50 g) was mixed with multifloral honey (7.5 g) and deionized water (12.5 mL), and freeze-dried bacterial culture (1 g, 3 × 10^9^ CFU) was added to the inoculated samples. Similar preparation was performed for additional ultrasound-treated pollen samples. The mixtures were sealed in glass vessels (up to 2/3 full) and initially incubated at 32 °C for 48 h, followed by fermentation at room temperature (25 °C) or cooling temperature (4 °C) for 4 weeks. After fermentation, the samples were dried at 45 °C for 48 h to achieve approximately 10–12% moisture content and then ground for analysis.	Fermented pollen exhibited increased polyphenol content and comparable antioxidant activity, while also accelerating the yeast growth rate. Additionally, it demonstrated a protective effect on Cu/Zn-superoxide dismutase 1 (sod1∆ yeast mutant) subjected to hydrogen peroxide-induced oxidative stress. Fermentation at a higher temperature (25 °C) resulted in a product more like bee bread, whereas the use of ultrasound and starter culture showed no apparent positive effects.	[45]
Improved bioactive composition and nutritional value	Enzymatic hydrolysis	Alcalase 2.4 L FG, Novozym 33095, Neutrase 1.5 MG, Protamex, Viscoflow MG, Suberase	Bee pollen was placed in a 250 mL flask and suspended in an equal volume of distilled water. Hydrolysis was carried out by adding each enzyme at a concentration of 0.05 units per gram of bee pollen to the suspension.	Proteases enhanced the protein content by approximately 13% to 18%, increased phenolics by 83% to 106%, and flavonoids by 85% to 96%. Antioxidant activity improved by up to 68%, and all essential amino acids were increased.	[11]
Antioxidant and antibacterial activities	Enzymatic hydrolysis	*Viscozyme^®^ L*, *Clara-diastase*, lipase, Protease, amyloglucosidase, cellulase	One gram of each bee pollen sample was moistened with 0.5 mL of sterile bi-distilled water and heated for 15 min at 121 °C. The optimal duration for enzymatic hydrolysis was evaluated by performing the process for 1 h, 2 h, 3 h, 4 h, and 5 h. The optimal enzyme amount was determined by using 50 µ, 100 µ, 150 µ, 200 µ, and 300 µL of each enzyme.	The results revealed a positive impact of enzymatic hydrolysis on the content and activity of biologically active compounds: total phenolic content increased by 1.1 to 2.5 times, total flavonoid content by 1.1 to 3.0 times, radical scavenging activity by 1.1 to 3.5 times, and antibacterial activity by 1.1 to 3.3 times.	[32]
Improved nutritional value	Enzymatic hydrolysis	Alcalase 2.4 L and Protamex	Bee pollen samples were incubated at pH 8.5 and 55 °C for Alcalase 2.4 L, and at pH 7 and 55 °C for Protamex. The hydrolysis process was initiated by adding the enzymes, with continuous gentle stirring, at concentrations of 0.55 U/g and 0.307 U/g of protein content in the substrate, respectively.	The results indicated that Alcalase 2.4 L exhibited a slightly higher proteolytic capacity (10.4%) compared to Protamex (9.3%). Enzymatic hydrolysis enhanced the commercial and nutritional value of the pollen proteins.	[1]
Antioxidant activity and improved nutritional value	Enzymatic hydrolysis	Gastrointestinal enzymes (pepsin and trypsin)	Defatted samples (14.5% protein) were dispersed in 0.1 M acetate buffer (pH 3) and 0.1 M potassium phosphate buffer and subjected to enzymatic digestion with pepsin and trypsin. The digestion progress was monitored at intervals of 1.0 h, 2.5 h, and 4.0 h, after which the process was halted by heating. The supernatants were then lyophilized for storage.	The results demonstrated that controlled enzymatic hydrolysis of pollen protein successfully produced bioactive peptides with high ACE-inhibitory and antioxidant activity, as evidenced by DPPH and FRAP assays, in the final product.	[71]
Antihypertensive activity (angiotensin-I converting enzyme inhibitory peptide)	Enzymatic hydrolysis	Alcalase	Bee pollen was dispersed in a 0.1 mol/L NaOH solution at a ratio of 1:20 (*w*/*v*). Alcalase (200 U/mL) was added to the solution, and hydrolysis was carried out at 60 °C for 4 h. The enzymatic hydrolysis process was halted by heating the solution to 90 °C for 10 min. The solution was then centrifuged at 6000× *g* for 10 min. The resulting supernatant was concentrated and lyophilized to produce an RBP protein hydrolysate.	The sample was dispersed in a 0.1 mol/L NaOH solution at a ratio of 1:20 (*w*/*v*). Alcalase (200 U/mL) was then added, and the mixture was hydrolyzed at 60 °C for 4 h. Enzymatic hydrolysis was stopped by heating the solution to 90 °C for 10 min, followed by centrifugation at 6000× *g* for 10 min. The supernatant was concentrated and lyophilized to yield an RBP protein hydrolysate.	[34]
Antioxidant activity	Enzymatic hydrolysis	Bromelain, aminopeptidase, and proline iminopeptidase of plant origin	Pollen samples (28% protein) were suspended in 5 volumes of distilled water and homogenized. The pH of the suspension was adjusted to 7.0 with NH_4_OH. Bromelain (8 mAU/g) was added to initiate digestion at 37 °C for 4 h, and the process was then halted by microwaving for 2 min. Further hydrolysis was performed by adding aminopeptidase (0.05 units/g substrate), proline iminopeptidase (0.03 units/g substrate), and additional aminopeptidase (0.2 units/g substrate), and incubating at 37 °C, pH 7.5, for 2 h with stirring. The hydrolysates were subsequently centrifuged at 6000× *g* for 30 min at 5 °C.	The degree of hydrolysis, total phenolic content, and protein content of the hydrolysates were as follows: DH ranged from approximately 20% to 28%, total phenolics ranged from 15 μg/mg to 27 μg/mg of sample powder, and proteins ranged from 163 μg/mg to 243 μg/mg of sample powder. Additionally, the hydrolysates demonstrated high antiradical scavenging activity, with DPPH inhibition ranging from 42% to 46%.	[35]
Improvement in food security (allergenicity alleviation)	Enzymatic hydrolysis	Cellulase, pectinase, and papain	10 mL of Millipore water and 5 g of bee pollen powder were mixed and vortexed for 5 min. For the two-enzyme-treated bee pollen (2E-BP) groups, 3000 U each of cellulase and pectinase were added. For the three-enzyme-treated bee pollen (3E-BP) groups, 3000 U each of cellulase, pectinase, and papain were added. All samples were adjusted to pH 4.0. Lyophilization was performed using a vacuum freeze dryer, and the samples were stored at −80 °C for further analysis.	Enzyme-treated bee pollen demonstrated potential to reduce the frequency of scratching in mice, improve histopathological injuries, lower serum IgE levels, and regulate bioamine production. Additionally, it could modulate metabolic pathways and gut microbiota composition in mice, further supporting its ability to alleviate allergenicity.	[33]
Antioxidant activity and improved nutritional value	Enzymatic hydrolysis	Pepsin	Preparation of pepsin extract of bee pollen: 20 g of bee pollen was mixed with distilled water acidified with concentrated HCl to achieve a pH of 2. Pepsin was added to reach a concentration of 1%, and the mixture was incubated at 37 °C for 48 h. Hydrolysis was terminated by boiling the mixture for 10 min. The resulting enzymatic extract was filtered and centrifuged. The supernatant was evaporated and then dried at 38 °C. Preparation of ethanol extracts of pepsin-digested bee pollen: The supernatant obtained after pepsin extraction of bee pollen was extracted with 200 mL of a 50% (*v*/*v*) ethanol aqueous solution for 60 min at room temperature, with frequent shaking.	Antioxidant activities were highest in the ethanol extracts of pepsin-digested bee pollen and were associated with the total content of phenolic and flavonoid compounds. Antioxidant activity was assessed using DPPH radical scavenging assays and Trolox equivalents antioxidant capacity assays.	[91]
Improvement in physicochemical properties	Physical treatments (ultrasonication, freeze-thawing and their combination), and enzymatic hydrolysis	Cellulase–pectinase–xylanase–papain, 4:2:1:3	Bee pollen was ground for 2 min, sifted through a 150-mesh sieve, and dispersed in petroleum ether for oil extraction. Various wall-breaking methods were employed: ultrasound treatment (U), temperature change (T), enzyme hydrolysis (E), combined temperature change and ultrasound (T + U), and combined enzyme hydrolysis and ultrasound (E + U). After treatment, the pollen was dispersed in water, centrifuged, and the supernatant was processed for protein precipitation. The resulting pellet was then dried using a freeze dryer. A control sample without wall-breaking treatment was also prepared.	Proteins extracted by the enzymatic hydrolysis method exhibited enhanced solubility, emulsifying, and gelation properties due to partial hydrolysis by protease. Additionally, combining ultrasound with freeze-thawing or enzymatic hydrolysis further improved protein yield and functional properties. This enhancement is primarily attributed to structural changes in the proteins induced by the cavitation effects of ultrasound.	[48]
Nutrient release improvement	Ultrasonication and high-shear technique	N/A	Each species of bee pollen (lotus, rape, apricot, wuweizi, and camellia) was mixed with ultrapure water and adjusted to pH 3.50. To prevent oxidation, specific amounts of vitamin C and E were added to each pollen type. The suspensions were then subjected to intermittent ultrasound treatment using an ultrasonic processor at 1000 W, 80 Hz, and 20 °C. Following ultrasonication, high-shear treatment was applied at 25,000× *g* for 50 s. The samples were then freeze-dried at −60 °C for 24 h until a constant weight was achieved, and subsequently stored at −18 °C until further use.	Amino acids were predominantly located within the cell walls of lotus, rape, apricot, wuweizi, and camellia bee pollen. After treatment, the pollen walls were thoroughly fragmented, releasing a significant amount of nutrients. Notable increases were observed in amino acids, fatty acids, proteins, crude fat, reducing sugars, β-carotene, calcium, iron, zinc, and selenium following wall disruption. The combination of ultrasonication and high-shear treatments was effective in breaking down bee pollen walls and releasing these nutrients.	[86]
Improved bioactive composition and nutritional value	Ultrasonic extraction	N/A	The ultrasonic extraction conditions for pollen were optimized using the RSM to maximize the total phenolic content. The experimental study employed a Box–Behnken design, which included variables such as ethanol ratio (30–70%), extraction time (5–15 min), and ultrasonic amplitude modulation (10–20%). The TPC of the obtained extracts was measured using the Folin–Ciocalteu method.	The optimal extraction conditions were predicted to be a 60.012% ethanol ratio, 11.054 min of extraction time, and 19.160% amplitude modulation. Under these conditions, the extraction was estimated to yield 9.572 mg of gallic acid equivalent (GAE)/g extract.	[43]
Antioxidant activity and improved nutritional value	Pollen pulverization, extraction with solvents (70% aqueous ethanol, ethanol, methanol, and water), and agitation, maceration, reflux, and sonication.	N/A	The extraction was carried out using 21 mL of solvent with a probe sonicator (Sonics Vibra Cell Model VCX130, Sonics, Newtown, CT, USA) operating at 130 W and 20 kHz. The amplitude was set at 100%, and the extraction was performed for 30 min in an ice bath. An extensive investigation was conducted to identify the most effective process for maximizing the extraction of components. This was to measure the total phenolic content, DPPH radical scavenging activity, and FRAP antioxidant activity in two bee pollen samples from Western Australia (Jarrah and Marri pollen).	The data indicate that non-pulverized bee pollen extracted with 70% aqueous ethanol using the agitation extraction method represents the optimal conditions for maximizing the extraction of phenolics and antioxidant compounds in these bee pollen samples.	[72]
Antimicrobial, antioxidant activity, and improved bioactive composition	Conventional and sonication extraction	N/A	For the conventional extraction method, bee pollen grains and powders were subjected to extraction with distilled water and 95% ethanol. The aqueous extraction was conducted using a 1:10 ratio of pollen to solvent, with heating at 45 °C for 3 h, followed by filtration and two additional extractions. Ethanol extraction was performed at room temperature for 24 h. The crude extracts from both methods were lyophilized and stored at −20 °C. For the sonication extraction, pollen was sonicated twice for 30 min each at 50 Hz, then filtered. The resulting filtrate was evaporated and lyophilized. Antioxidant activity was evaluated using the DPPH free radical scavenging assay.	None of the bee pollen crude extracts demonstrated antibacterial activity against the tested pathogenic bacteria, including *Bacillus cereus*, *E. coli* strains, *methicillin-resistant S. aureus*, *Salmonella typhi*, *Shigella dysenteriae*, *S. aureus*, and *Vibrio cholerae*. However, the ethanolic extract of bee pollen grains obtained through the conventional method showed the highest antioxidant activity (41 ± 3) mg GAE/g extract, *p* < 0.05. For phenolic content, the aqueous extract of bee pollen grains using the conventional method had the highest total phenolic content at (103 ± 2) mg GAE/g extract. The ethanolic extract of bee pollen powder, also using the conventional method, exhibited the highest flavonoid content at (56 ± 5) mg quercetin equivalents/g extract.	[92]
Improved phenolic content, antioxidant activity, and bioactive profile	Green extraction techniques (microwave- and ultrasound-assisted) vs. conventional extraction (maceration and magnetic stirring)	N/A	Maceration extraction: Pollen samples (2 g) were combined with 40 mL of 80% ethanol in an Erlenmeyer flask and incubated in the dark for 72 h with occasional agitation. The resulting mixture was vacuum-filtered, evaporated at 40 °C, and freeze-dried for storage at −20 °C. Magnetic stirring extraction: 2 g of each sample was mixed with 40 mL of 80% ethanol and agitated with a magnetic stirrer for 6 h. The mixture was vacuum-filtered, re-extracted with an additional 40 mL of solvent, combined, evaporated at 40 °C, lyophilized, and stored at −20 °C. Ultrasound-assisted extraction: 2 g of bee pollen sample was mixed with 40 mL of 80% ethanol and subjected to ultrasonic homogenization at 400 W for 20 min. The extracts were vacuum-filtered, evaporated at 40 °C, lyophilized, and stored at −20 °C. Microwave-assisted extraction: 2 g of bee pollen sample was mixed with 40 mL of 80% ethanol in a round-bottom angled three-neck flask and subjected to microwave extraction at 200 W for 10 min. The extracts were then processed similarly to other methods and stored at −20 °C.	The microwave-assisted extraction produced the highest total phenolic content at 28 mg/g and flavonoid content at 8 mg/g, outperforming both the magnetic stirring and maceration methods. The extracts contained a total of twenty-six bioactive compounds, which included thirteen phenolics and thirteen phenylamides. While the extraction technique had a minimal impact on the chemical diversity of the extracts, green extraction methods significantly increased the yields of bioactive compounds, with phenolics increasing by 40% to 60% and phenylamides by up to 200%. The antioxidant activity assays confirmed that bee pollen is a potent source of antioxidants, with the most bioactive extracts being those obtained through green extraction techniques.	[2]
Antimicrobial activity and improved bioactive profile and nutritional value	Ultrasonic extraction method and deep eutectic solvents (DESs)	N/A	In the study, five yeast-like fungi and ten bacterial strains (including five Gram-positive and five Gram-negative bacteria) were utilized. Stock cultures were maintained at −20 °C and subsequently inoculated onto appropriate media, followed by incubation at 37 °C for 24 h. The effects of various process variables, such as the molar ratio of DES (1, 1.5, and 2), sonication time (15 min, 30 min, and 45 min), and ultrasonic power (90 W, 135 W, and 180 W), were examined. These factors were analyzed using RSM to assess their impact on the total content of individual amino acids, organic acids, and phenolic compounds in the samples.	The study identified optimal extraction conditions as a molar ratio of 2, sonication for 45 min, and an ultrasonic power of 180 W, achieving a determination coefficient (*R^2^*) of 0.84. When compared to the control extracts obtained through maceration with ethanol, the DESs produced higher levels of total individual amino acids and organic acids. Furthermore, DESs were more effective in extracting myricetin, kaempferol, and quercetin at higher concentrations than the control. Antimicrobial tests demonstrated that DESs exhibited broad-spectrum antibacterial activity against all tested bacterial strains. However, their inhibitory effect on yeast-like fungi samples was minimal.	[44]
Improved phenolic content and reduction of oxidative stress and steatosis in hepatic cells	Ultrasonic extraction	N/A	Honeybee pollen extracts were prepared using ultrasonic extraction at a frequency of 37 kHz and 240 W, with one gram of fresh honeybee pollen in 10 mL of analytical grade absolute ethanol, at room temperature (25 °C) for 10 min. After extraction, the mixture was centrifuged at 3130× *g* for 5 min. The supernatants were pooled to a final volume of 50 mL (1 g/50 mL) and stored at −80 °C in darkness until further analysis. The total polyphenol content of the extracts was quantified using the Folin–Ciocalteu method, with results expressed in mg GAE/100 g of bee pollen. The flavonoid content was determined via the AlCl3 method and reported as milligrams of quercetin equivalents per 100 g of bee pollen (mg QE/100 g). Selected BPE samples were then tested for their potential to reverse steatosis in an *in vitro* model using Hepa1-6 cells.	A statistically significant positive correlation was observed between the total phenolic content and the antioxidant capacity of the bee pollen extracts. These extracts were found to protect Hepa1-6 cells from oxidative damage induced by free radicals generated by 2,20-azo-bis(2-amidinopropane) dihydrochloride (AAPH). The protective effect is likely due to the phenolic compounds’ potent ability to scavenge free radicals, thereby safeguarding liver cells from chemically induced damage. Furthermore, the bee pollen extracts demonstrated a reduction in lipid accumulation in a cellular steatosis model, suggesting their potential to mitigate fatty liver conditions.	[93]
Improvement in digestibility and bioavailability of intracellular bioactive compounds	Protamex hydrolysis and ultrasonication	ProtamexTM protease	The wall-disruption variations of rape bee pollen treated with protamex hydrolysis, ultrasonication, and a combination of both methods were thoroughly investigated. The effects of these treatments on the pollen’s structure were evaluated using specific surface area, particle size distribution, scanning electron microscopy (SEM), and transmission electron microscopy. For the protamex hydrolysis treatment, dried pollen particles (25.0 g) were mixed with Milli-Q ultrapure water (100 mL), and the pH was adjusted to 8.0 using an NH4OH solution. ProtamexTM protease (0.5 g) was then added, and the mixture was incubated at 48 °C for 48 h on a mechanical shaker. For the ultrasonication treatment, a larger batch of dried pollen particles (200.0 g) was suspended in Milli-Q ultrapure water (800 mL) and divided into six 100 mL samples. Each sample was subjected to varying durations of sonication (ranging from 1 h to 6 h) at 48 °C, using an ultrasonic processor (DCTZ-1000, Multi Measuring Instruments Co., Ltd., Tokyo, Japan) operating at 1000 W and 80 kHz frequency. In the combined treatment, the procedure began similarly to the PH method, where ProtamexTM protease (4.0 g) was added to the mixture before sonication. The combined treatment aimed to leverage the synergistic effects of enzymatic hydrolysis and mechanical disruption from ultrasonication to enhance the wall-breaking efficiency. These different methods provided insights into the impact of various wall-disruption techniques on the physical properties and structural integrity of the bee pollen, potentially leading to improvements in nutrient release and bioavailability.	The findings highlight the effectiveness of the combined protamex hydrolysis and ultrasonication treatment in fully disrupting the pollen coat and cell walls, which could be critical for enhancing the bioavailability of nutrients and bioactive compounds in rape bee pollen. This method may offer significant advantages for applications requiring maximal extraction of pollen constituents.	[39]
Improvement in digestibility and bioavailability of intracellular bioactive compounds	Wall-disrupted treatment with a combination of ultrasonication and high-shear technique (US-HS)	N/A	The wall-disruption treatment described involved a series of steps aimed at breaking down the cell walls of various bee pollen samples to enhance nutrient release and bioavailability. The procedure started with dissolving 80 g of each pollen sample in 800 mL of ultrapure water, followed by pH adjustment to 3.50 using phosphate buffer. To prevent oxidation, vitamins C and E were added to each solution. The samples were then subjected to high-shear treatment in an ice bath for 50 s, after which they underwent intermittent ultrasound treatment using an ultrasonic processor set to 1000 W, 80 Hz frequency, and 20 °C. The duration of ultrasonication varied depending on the pollen type: 4 h for rape bee pollen, 1 h for lotus bee pollen, 16 h for camellia bee pollen, 13 h for wuweizi bee pollen, and 8 h for apricot bee pollen. Following the ultrasonication, the pollen solutions were vacuum freeze-dried to a moisture content of ≤8% and stored at −18 °C until further use.	The study revealed that after both *in vitro* and *in vivo* digestion, intact bee pollen grains remained largely unbroken, whereas fragments of wall-disrupted bee pollen persisted. *in vivo* digestion experiments conducted on mice demonstrated that wall-disrupted bee pollen was more efficiently emptied from the gastrointestinal tract compared to unbroken pollen. Dynamic *in vitro* digestion further indicated that the digestibility of proteins and crude fat in wall-disrupted bee pollen significantly increased, exceeding 80%. Additionally, the release rates of amino acids and reducing sugars in wall-disrupted samples were approximately 1.5 and 2 times higher, respectively, compared to unbroken pollen samples. This suggests that wall-disruption treatments enhance the bioavailability of nutrients in bee pollen.	[42]
Improvement in physicochemical properties	Ultrasonication and hydro-alcoholic extraction	N/A	Different ultrasonication times ranging from 15 min to 45 min were employed to study the disintegration of pollen walls. Additionally, varying amounts of ethanol in the hydro-alcoholic solvent, from 0% to 100% (*v/v*), were investigated to optimize the extraction of the main bioactive compounds from the pollen.	The most desirable pollen extract was obtained using 95% (*v/v*) ethanol as the solvent and ultrasonication for 44 min. This extract exhibited minimal pH (5.95), particle size (229 nm), and polydispersity index (0.182), along with a maximum zeta potential of −14.9 mV. It also demonstrated high antioxidant activity (87% inhibition) and total phenol content (200 mg/mL). Gas chromatography analysis revealed the presence of two significant phenolic compounds, carvacrol and thymol, known for their unique biological and antimicrobial activities.	[94]
Antioxidant activity and improved nutritional value	Ultrasonic and ball-milling treatment	N/A	Rose bee pollen underwent two different treatments: ball-milling and ultrasonic treatment. For ball-milling, rose bee pollen was subjected to 3 min of ball-milling at 25 Hz, resulting in ball-milling-treated rose bee pollen. For ultrasonic treatment, 40 g of rose bee pollen was treated with distilled water using an ultrasonic processor for 4 h at 25 °C. The treated pollen was then vacuum freeze-dried to produce ultrasonic-treated rose bee pollen. The effects of these treatments on aging mice were evaluated by measuring various parameters, including organ indexes, nitric oxide levels, catalase and superoxide dismutase activity, as well as glutathione and malondialdehyde levels.	The treatment of rose bee pollen through wall-breaking methods, including ball-milling and ultrasonic treatment, enhanced its antioxidant properties as demonstrated by improved scavenging effects against DPPH and ABTS radicals and increased ORAC. In aging mice, this treatment led to the following: improved organ recovery, enhanced activities of superoxide dismutase and catalase, reduced levels of malondialdehyde. Additionally, eight compounds were identified from the ethanol extract of rose bee pollen, including isorhamnetin 3-O-diglucoside and N′, N″, N‴-dicaffeoyl p-coumaroyl spermidine, which are notable for their potential biological activity.	[41]
Improvement in antioxidant properties	Microwave- and ultrasound-assisted extraction	N/A	For bee pollen extraction, two methods were used: Microwave-assisted extraction: A 1350 W microwave with a modified frequency of 60 Hz was employed. Extractions were conducted using 1 g of pollen with varying ethanol volumes (10 mL and 50 mL) and electrical powers (135 W, 405 W, and 945 W) for durations of 6 s, 12 s, and 24 s. Temperature was monitored during each treatment. The resulting extracts were filtered and stored at −20 °C until analysis. Ultrasound-assisted extraction: 1 g of pollen was mixed with 10 mL of food-grade ethanol. Ultrasound was applied using an ELMA 30H bath operating at a frequency of 35 kHz and a power of 250 W for 15 min. The mixture was then centrifuged, and the supernatant was filtered.	The total polyphenol content was higher in the extracts obtained by microwave-assisted extraction, while the antioxidant activity was greater in the extracts obtained by ultrasound-assisted extraction.	[70]
Improvement in nutrient profile, physicochemical, and biological (antimicrobial) properties	Planetary ball-milling and ultrasonication	*E. coli*, *S. aureus*, *Listeria monocytogenes*, *Pseudomonas aeruginosa*, *Candida albicans*, and *S. cerevisiae*	The effectiveness of various pretreatments, including ball-milling and ultrasonication, was evaluated based on their impact on nutritional values, physicochemical properties, and antimicrobial activity of the pollen.	Ball-milling treatment significantly increased the protein, sucrose, glucose, fructose, lipids, moisture, and ash content in the amorphous pollen powder, producing monodispersed particles with a prolate–oval shape. Compared to untreated pollen, these increases were up to 58%, 88%, 41%, 275%, 13%, 53%, and 66%, respectively (*p* < 0.05). Elemental analysis showed higher weight percentages of potassium, calcium, iron, nickel, selenium, and barium in the treated pollen powder. Gas chromatography–mass spectrometry (GC-MS) analysis revealed an increase in the number of bioactive compounds from 64 in untreated pollen to 119, 136, and 161 in pollen treated with ball-milling, ultrasonication, and ultrasonication–ball-milling, respectively. Additionally, pollen pretreated with ultrasonication–ball-milling exhibited the highest antioxidant activity (94% inhibition), total phenol content (45 mg/mL), and antimicrobial activity against various microorganisms, *including E. coli* (20 mm), *S. aureus* (23 mm), *L. monocytogenes* (22 mm), *P. aeruginosa* (8 mm), *C. albicans* (21 mm), and *S. cerevisiae* (20 mm), based on the diameter of the clear zone.	[69]
Antioxidant activity	Ultrasound and solvent extraction	N/A	The pollen pellets were suspended in water-miscible solvents and subjected to ultrasound at 41 °C for 90 min. After centrifugation, antioxidant assays were conducted on the obtained extract. Methanolic extracts were prepared by sonicating pollen in methanol (MeOH), followed by evaporation and vacuum-drying overnight. The residues were then dissolved in MeOH for analysis.	The Mimosa pollen sample exhibited the highest antioxidant activity. Total polyphenolics, flavanols, and flavones were quantified, showing a strong correlation between antioxidant activity and total phenolics.	[66]
Antioxidant activity and protective effect on DNA and lymphocytes damage response to oxidative stress.	Ultrasound-assisted ethanol extraction	N/A	*A. arguta* pollen was extracted using four methods: A, B, C, and D. Method A involved ultrasound-assisted extraction with water, where 20 g of pollen was treated with 300 mL of distilled water in an ultrasonic device at 40 kHz frequency and 700 W for 2 h at 70 °C. The resulting supernatant was filtered and concentrated to obtain AAPE. Method B employed heat reflux extraction with water, using heat refluxing at 70 °C for 2 h. Method C utilized 75% ethanol for ultrasound-assisted extraction, while Method D used 75% ethanol for heat reflux extraction. The extracted AAPE from each method was stored at 4 °C for analysis.	*Actinidia arguta* pollen extract obtained through various extraction methods demonstrated protective effects against DNA oxidative damage and strong cytoprotective effects on mouse lymphocytes. Method C yielded the extract with the highest total phenolic content (15.1 mg GAE/g ± 0.3 mg GAE/g) and exhibited notable ferrous ion-chelating ability (0.37 mg Na2EDTA/g ± 0.02 mg Na2EDTA/g), DPPH⋅ scavenging activity (IC_50_ = 0.14 mg/mL ± 0.04 mg/mL), and FRAP (7.1 mg Trolox/g ± 0.3 mg Trolox/g).	[38]
Improvement in nutrient profile	Ultrasound-assisted extraction	N/A	The extraction from defatted pollen was conducted by varying four parameters: ultrasonic amplitude (20%, 60%, and 100%), solid/liquid ratio (10 g/L, 20 g/L, and 30 g/L), temperature (35 °C, 50 °C, and 65 °C), and extraction time (10 min, 20 min, and 30 min).	The extracts were analyzed for extraction yield (%), total phenolic content, and total flavones content. The extracted oil was also analyzed for fatty acid composition, revealing myristic acid (159 µg/g) and cis-14-pentadecenoic acid (106.6 µg/g) as the most abundant. The optimal extraction conditions were determined to be 100% amplitude of ultrasonic treatment, a 30 g/L solid/liquid ratio, 40.85 °C, and 14.30 min. Under these conditions, the extraction yielded 366 mg GAE/L, 592.2 mg QE/g, and an extraction yield of 1.92%.	[13]
Improvement in nutrient profile and antioxidant activity	Microwave-drying (MWD), microwave-assisted vacuum-drying (MW-VD), hot-air-drying (HAD), vacuum-drying (VD), and freeze-drying	N/A	Fresh bee pollen samples underwent dehydration using different drying techniques to lower their moisture content to under 8%. The methods applied were conventional hot-air-drying (HAD), vacuum-drying (VD), microwave-drying (MWD), microwave-assisted vacuum-drying (MW-VD), and freeze-drying. MWD and MW-VD processes were executed at power settings of 300 W, 450 W, 600 W, and 900 W. The MW-VD setup included a standard microwave oven with a 900 W output, a polycarbonate vacuum chamber positioned in the center of the turntable, and a vacuum pump. A condenser was integrated into the vacuum line to collect water vapor released during the drying process. Prior to activating the microwave at the desired power levels, 45 g of fresh bee pollen was evenly distributed on a petri plate inside the container, and the necessary vacuum was applied. The surface temperature of the dried pollen samples was measured at two points using an infrared thermometer after each MWD and MW-VD session. A uniform amount of pollen (45 g) was used for all drying experiments. HAD and VD were conducted at three temperature settings (35 °C, 50 °C, and 65 °C), while freeze-drying was performed at a vacuum pressure of 0.1 mbar and a chamber at −50 °C.	Drying pollen at 35 °C preserved vitamin C levels nearly equivalent to fresh pollen, while other methods led to significant reductions ranging from 14.3% to 61.3%. Vitamin E content in the dried pollen varied between 71% and 87%. In addition to vitamin C, lower power levels during microwave-drying provided results that were equal to or better than those obtained through hot-air and freeze-drying. The study suggests that pollen dried with microwave-assisted vacuum-drying demonstrated superior antioxidant activity compared to hot-air-dried samples, irrespective of the pressure or power settings used.	[15]
Improvement in nutrient profile: amino acids (FAAs), *α*-dicarbonyls (α-DCs), and volatile compounds (VOCs)	Pulsed vacuum-drying (PVD), freeze-drying, infrared-drying (IRD), hot-air-drying (HAD) and sun drying (SD)	N/A	The pollen samples were dried using five distinct methods: PVD, freeze-drying, IRD, HAD, and SD, targeting a final moisture content of approximately 5%, suitable for long-term storage. Drying temperatures for PVD, IRD, and HAD were maintained at 45 °C to avoid the formation of Maillard reaction products. The PVD process alternated between vacuum and atmospheric pressure for 12 min and 3 min, respectively. Freeze-drying involved an initial freezing step at −40 °C for 16 h, followed by drying at 25 °C and 50 Pa. The IRD process was carried out using a YXD-F9 (Nabertherm GmbH, Lilienthal, Germany) infrared-drying oven operating at 1000 W. HAD was conducted at 45 °C with an airflow rate of 3 m/s, while SD involved drying fresh RBP samples at room temperature (25 °C).	The results indicated that freeze-drying led to a significant increase in the release of essential amino acids (EAAs) compared to fresh bee pollen, whereas sun drying (SD) caused the greatest reduction. Glucosone was identified as the predominant α-dicarbonyl compound in raw bee pollen (RBP), with pressure vacuum-drying (PVD) resulting in the highest loss. Aldehydes were the main volatile compounds detected in both RBP and SD samples, with SD samples showing a higher presence of newly formed volatile substances, particularly aldehydes, compared to the other drying methods. Overall, freeze-drying and PVD were identified as effective techniques for preserving the quality of RBP during drying.	[51]
Product quality and nutrient profile improvement	Hot-air (HAD) and vacuum (VD) drying, microwave (MWD) and microwave assisted vacuum-drying (MW-VD), and freeze-drying	N/A	Fresh bee pollen samples were dehydrated using several methods to reduce moisture content below 8%. The techniques included freeze-drying, traditional hot-air-drying, vacuum-drying at varying pressures and temperatures, microwave-drying, and microwave-assisted vacuum-drying at different power settings. The microwave-assisted vacuum-drying setup included a microwave, a vacuum chamber, a condenser, and a vacuum pump. Samples were placed in the vacuum chamber inside the microwave oven, with a vacuum pump creating the necessary pressure, and a condenser used to collect water vapor at low temperatures.	Samples exposed to MWD and MW-VD at 600 W and 900 W experienced a significant decrease in diastase number, with microwave treatment at 900 W causing the largest reduction, approximately 72–76%. In contrast, higher retention of enzyme activity, ranging from 87 to 93%, was observed at 50 °C during hot-air and vacuum-drying. Proline and HMF levels were influenced by the power levels used in MWD and MW-VD. Notably, a significant reduction in proline content occurred only in samples treated with the highest microwave power. Except for the highest power level, MW-VD led to a marked increase in HMF content, ranging from 5 mg/kg to 9 mg/kg, whereas hot-air and vacuum-drying methods maintained HMF content similar to that of fresh pollen, aside from treatments at the highest power.	[63]
Improvement in biochemical profile	Oven-drying and lyophilization	N/A	Fresh bee pollen was dried in an oven (Nuve EN 400; Nuve, Turkey) at 50 °C for 8 h to obtain oven-dried bee pollen samples. For freeze-drying, the pollen was processed using an Operon freeze dryer at −50 °C and 0.1 mbar pressure for 24 h.	Pollen samples were assessed for moisture, total lipid, protein, pH, and total phenolic content. The results indicated moisture levels ranging from 6.2% to 20.6%, total lipid content between 5.0% and 5.6%, protein content between 16.8% and 1.5%, pH values from 4.08 to 4.33, and total phenolic content between 15.2 mg GAE/g and 22.73 mg GAE/g. All samples were found to be rich in squalene and methyl octadecanoate. It was observed that drying methods utilizing milder conditions, such as lyophilization, cause less damage to the bioactive components of bee pollen compared to conventional methods.	[95]
Product quality and nutrient profile improvement	Tray-drying	N/A	A 1 kg sample was gathered from each apiary and brought to the drying laboratory in sealed glass jars at ambient temperature. The bee pollen was cleaned to remove debris, including chaff, bees, and leaves. A total of 500 g of the cleaned pollen was allocated for hot-air-drying, while the remaining portion was set aside for chemical analysis. The pollen pellets were dehydrated using a tray dryer with hot air, avoiding air recirculation, at 35 °C and 45 °C, to reach a final moisture content below 12%. In Bogotá, the relative humidity generally ranges between 60% and 70%, but this was not controlled within the dryer. The drying process was conducted with consistent parameters: (1) an air velocity of 3 m/s, determined by the equipment configuration and fan; (2) a sample thickness of 0.02 m due to the amount of sample used (250 g per tray); and (3) a drying area of 0.07 m^2^ per tray.	The results indicate that drying bee pollen at 45 °C shortens the drying time (156–198 min), reduces moisture content (7–8%), and lowers water activity (0.3). However, this temperature also leads to increased losses of carotene and vitamin C. Despite these changes, the drying temperature does not significantly impact the protein, fiber, or ash contents.	[67]
Improvement in biochemical properties	Infrared-radiation drying	N/A	Infrared-drying experiments utilized a moisture analyzer equipped with a 250 W halogen lamp. Approximately 25 g of samples were spread evenly over the pan to a thickness of about 4 mm. The drying process was carried out at power levels ranging from 50 W to 88 W, with power controlled by the equipment unit and monitored using an energy meter. During the drying trials, the pan was removed every 10 min to record weight loss with a digital balance accurate to ±0.01 g. All experiments were conducted in triplicate until the bee pollen achieved a final moisture content of (0.081 ± 0.003) g water/g dry solid. The dried samples were then vacuum-packed using composite film with an aluminum barrier layer to protect against oxygen and water vapor.	Infrared power had a significant effect on drying time and quality, particularly on color. Increasing the infrared power from 50 W to 88 W reduced the drying time from 170 min to 50 min. Higher infrared power levels resulted in more pronounced morphological changes on the surface of bee pollen grains. Bee pollen dried at 50 W maintained better quality compared to samples dried at higher power levels.	[14]
Improvement in quality characteristics, physicochemical properties, morphological structure, and organoleptic characteristics	Hot-air-drying	N/A	Hot-air-drying experiments were carried out at five different temperatures (40 °C, 45 °C, 50 °C, 55 °C, and 60 °C) using a cabinet dryer. The drying air temperatures were monitored with a digital temperature controller unit that had an accuracy of ±0.5 °C, while air velocity was measured using an anemometer with a sensitivity of ±0.1 m/s. Prior to the drying trials, the dryer was operated empty for 30 min to stabilize conditions. Bee pollen samples (25 g) were evenly distributed in a single layer, approximately 0.004 m thick, on stainless steel drying trays. The trays were removed every 10 min, and moisture loss was measured using a digital balance with an accuracy of ±0.01 g. The trials were performed in triplicate until the bee pollen achieved a final moisture content of (0.088 ± 0.002) g water/g dry solid.	The effective moisture diffusivity values ranged from 1.38 × 10^−10^ m^2^/s to 4.00 × 10^−10^ m^2^/s, with an activation energy of 42.96 kJ/mol. Drying temperature affected the protein, fat, total carbohydrates, and vitamin C content in bee pollen. Dried samples exhibited high solubility indices and lower L* and b* values compared to fresh pollen. The total color difference (ΔE) was minimized for pollen dried at 40 °C. Higher drying temperatures led to more pronounced morphological changes on pollen surfaces. Bee pollen dried at 40 °C received the highest sensory scores and preserved quality better than samples dried at 45 °C, 50 °C, 55 °C, and 60 °C. Therefore, hot-air-drying at 40 °C is recommended for optimal bee pollen drying.	[96]
Improvement in physical, chemical, and biological parameters and the microbiological quality	Lyophilization, electric oven-drying with forced air circulation	N/A	Lyophilization: samples were first frozen at −40 °C and subsequently dehydrated using a vacuum lyophilizer for a duration of 18 h. Electric oven-drying with forced air circulation: after thawing at room temperature, the samples were dried in an electric oven at 42 °C for 24 h to 52 h, achieving a moisture content like that of lyophilized samples (6 g/100 g to 8 g/100 g).	The levels of glucose and fructose stayed constant, whereas protein and lipid contents varied. Oven-dehydrated samples with forced air circulation had significantly lower vitamin E content compared to those that were lyophilized. Results for the vitamin B complex, minerals, and microbiological indicators were inconclusive. Positive correlations were observed between color parameters (L* and b*) and total phenolic content, as well as between phenolic content and antioxidant and antimicrobial activities. These findings indicate that lyophilization could serve as a viable alternative, producing bee pollen with improved biological activity.	[97]
Changes in chemical content of pollen (protein, fat and water content, ash, carbohydrate, and dissolved protein)	Sun-dried pollen and oven-dried pollen	N/A	Pollen samples dried for 12 h were categorized into two treatments: P0 (sun-dried pollen), with no further drying, and P1 (sun + oven-dried pollen), which underwent additional drying in an oven at 60 °C for 4 h. Each treatment, consisting of 100 g samples, was analyzed for protein, fat, moisture, ash, and carbohydrate content. Protein levels were quantified using the Lowry method and measured with a spectrophotometer.	The study revealed significant differences (*p* < 0.01) in protein, fat, and ash contents across the drying methods, highlighting their effect on nutrient composition. Notably, oven-drying led to reduced water content, minimizing the risk of pollen spoilage, and resulted in higher levels of proteins, fats, and carbohydrates, which are advantageous for honeybee colonies.	[98]
Product quality and nutrient profile improvement	Hot-air-drying	N/A	Fresh rape bee pollen samples were spread in a single layer on a feeder tray with dimensions (200 × 150 × 10) mm, having a layer thickness of 8 mm and an average weight of (200 ± 5) g. Hot-air-drying was performed at 40 °C, 50 °C, 60 °C, and 70 °C, using an oven with an air velocity of 1 m/s at the pollen surface. Samples were collected every 20 min until they reached a final moisture content of approximately 6%. After drying, the samples were cooled to room temperature, placed in polythene bags, and stored in a desiccator at room temperature.	Increasing the drying temperature from 40 °C to 70 °C led to a 65% reduction in drying time. Hot-air-drying resulted in decreased L* and b* values while increasing a* values. Higher drying temperatures also caused a rise in the browning index and 5-HMF content. Antheraxanthin content increased by 230% at 70 °C, whereas lutein and zeaxanthin decreased by 74% and 81%, respectively, compared to fresh pollen. The levels of 3-deoxyglucosone, 1-deoxy-2,3-pentosulose, antheraxanthin, and lutein were linked to color deterioration during the drying process for rape bee pollen.	[47]
Improvement in microbiological, structural and physicochemical properties	Conventional hot-air-drying	N/A	Bee pollen was dried in a conventional hot-air oven (Heraeus Vötsch VMT 07/35, Heraeus-Vötsch, Burladingen, Germany) at of 40 °C, 50 °C, and 60 °C, with a constant air flow of 3 m/s. Each trial, conducted in triplicate, used 1.5 ± 0.1 kg of bee pollen. Preliminary tests established that 6 h of drying at these temperatures was sufficient to achieve a final moisture content below 8%.	The results showed that bee pollen dried at 40 °C and 50 °C underwent a significant increase in acidity, likely due to residual microbiological load. All thermal treatments, however, led to a notable rise in flavonoids, phenolics, and antioxidant activity. Despite this, carotenoids decreased because of the drying temperature. Microscopy revealed minor structural degradation in the bee pollen, which may have contributed to the release of bioactive compounds and an enhanced antioxidant capacity. Overall, a comprehensive ranking method identified 60 °C as the optimal drying temperature for bee pollen.	[99]
Change in color, and improvement in total phenolic content, total flavonoid content, and antioxidant capacity	Hot-air and vacuum-drying	N/A	Pollen drying was carried out using a cabinet dryer at 40 °C, 45 °C, and 50 °C. The cabinet dryer, as detailed by Doymaz, operated with a constant air velocity of 2.0 m/s above the product. Vacuum levels were controlled by a pump with an ultimate pressure of 60 mbar and a pump speed of 2 L/s. The pollen samples were evenly spread in a round glass tray with a diameter of 15 cm and dried once the dryer reached steady-state conditions. Moisture loss was monitored every 15 min using an analytical balance with 0.1 mg precision. Drying continued until the pollen’s moisture content reached 8%. After drying, the samples were stored in glass jars at −18 °C for subsequent quality analysis.	After the drying processes, the total phenolic content, total flavonoid content, and antioxidant activity of bee pollen decreased. Vacuum-drying at 45 °C yielded higher total phenolic and total flavonoid content compared to other methods. Additionally, the total color change of bee pollen dried under vacuum conditions was lower than that of pollen dried using hot-air conditions.	[100]
Changes in drying kinetics, physicochemical properties, and microstructure	Pulsed vacuum-drying	N/A	Pollen samples were dried using a pulsed vacuum dryer under atmospheric pressure (101 kPa) and a vacuum of 6 kPa, with a pulsed drying ratio of 12 min on and 3 min off to ensure optimal quality. Approximately 900 g of samples were divided into five groups of about 180 g each. These groups were further split into three sets of 60 g and dried from 35 °C to 75 °C. To standardize moisture content, all pollen underwent vacuum freeze-drying for 24 h following the pulsed vacuum-drying. The dried samples were then ground, sieved through a 60-mesh screen, and stored at −18 °C for further analysis.	As the drying temperature increased from 35 °C to 75 °C, drying time was reduced by 233 min, but both antioxidant levels and lipid oxidation increased. Temperatures above 55 °C led to significant accumulation of HMF and α-dicarbonyls (α-DCs), with a notable decrease in amino acid content, particularly essential amino acids, by more than 32.2%. The microstructural analysis revealed accelerated water migration and intensified Maillard reactions at higher temperatures. The study investigated the quality changes in bee pollen under pulsed vacuum-drying, examined the pathways for HMF and α-DCs, identified 3,4-dideoxyglucosone (3,4-DGE) as a safety indicator, and recommended not exceeding 55 °C to maintain pollen quality.	[57]
Improvement in the bioactive profile	Freeze-drying, microwave-assisted drying (MWD) and classic hot-air-drying	N/A	Commercial bee-collected pollen was dried at 32 °C for 24 h using the NTW 100 cool-air dryer, resulting in a residual moisture content of 7%. Freeze-drying was applied to both treated and untreated pollen samples using a Heto PowerDry^®^ LL1500 lyophilizer (Thermo Fisher Scientific, Waltham, MA, USA) with samples stored at −20 °C until analysis. For MWD, pollen from chestnut, willow, and ivy was subjected to microwave-drying at 50 mbar and 150 W power for varying durations.	Drying and storage affected the chemical composition of pollen, with variations observed across species. MWD was most effective in preserving flavonoids in chestnut pollen for up to six months. Although all drying methods decreased flavonoid levels in willow pollen, MWD retained the highest concentration after six months. Neither freeze-drying nor MWD caused flavonoid loss in ivy pollen during storage. Rutin content, which was highest in freeze-drying willow samples after six months, remained stable. Both freeze-drying and MWD techniques effectively maintained the amino acid-related quality of bee pollen over six months of storage.	[53]
Improvement in bioactive profile and biological properties	Conventional drying and lyophilization	N/A	Sample preparation: Bee pollen was divided into two portions for drying; Portion 1: Frozen at −40 °C and then dehydrated for 18 h using a vacuum lyophilizer; Portion 2: Thawed at room temperature and then dried in an electric oven with forced air circulation (ESA 1368, Sercon, Multi Measuring Instruments Co., Ltd., Tokyo, Japan) at 42 °C. The drying time varied between 24 to 52 h, depending on when the moisture content reached 6–8%. Post-drying: Samples were vacuum-packed in food-grade polyethylene bags, stored at room temperature, and protected from light. Analysis: Analysis was conducted within 30 days of storage. Methanolic extracts of the bee pollen were prepared for further examination.	- Lyophilization resulted in the following: - Higher concentrations of flavonoid and phenolic compounds. - Stronger antimicrobial activity against Gram-negative and Gram-positive bacteria compared to other methods. - Superior antioxidant activity. - Maintenance of antimutagenic properties across all samples, with some types of bee pollen being more effective in reducing gene conversion and mutant colonies. - Other Preservation Methods: - Showed less pronounced differences in antimicrobial activity against yeasts. - Were less effective in preserving flavonoid and phenolic contents compared to lyophilization. Overall, lyophilization is highlighted as the most effective method for preserving the bioactive compounds and biological properties of bee pollen.	[87]
Changes in functional compounds profile and antioxidant activity	Solar dehydration	N/A	The harvested raw bee pollen was dried using a solar dehydration system configured as a greenhouse. The pollen was spread in a thin layer across trays, with approximately 700 g of pollen per tray and a total of 8 trays in the system. The solar-drying process was conducted over two consecutive days, from 7:00 a.m. to 3:00 p.m., to maximize exposure to sunlight. Pollen samples were collected hourly from 0 h to 8 h throughout the drying period. After drying, the samples were stored in hermetically sealed glass jars for subsequent analysis at the Instituto de Ciencia y Tecnología de Alimentos (ICTA) at the Universidad Nacional de Colombia.	The average carotenoid content in dried bee pollen was 0.9 mg β-carotene/g, while the phenolic compound content was 16 mg GAE/g. These levels remained relatively stable compared to the initial raw bee pollen. The antioxidant activity of raw bee pollen, measured at 0.85 mmol Trolox/g, was also preserved after drying in the solar dehydration system. The study concludes that solar-drying has minimal impact on the bioactive compound content of bee pollen, making it a viable alternative to traditional drying methods. This approach offers lower energy costs while maintaining product quality.	[88]
Changes in chemical content and morphological characteristics of Pollen	Microwave-drying	N/A	Bee pollen samples were dried using a domestic digital microwave oven with the following specifications: 230 V, 50 Hz, 2450 MHz frequency, and a cavity size of (505 × 574 × 376) mm. The microwave oven could operate at power levels ranging from 180 W to 900 W. The drying process involved placing bee pollen samples in Petri dishes and applying microwave power at levels of 180 W, 360 W, 540 W, 720 W, and 900 W. During the drying process, weight loss and surface temperatures were monitored until the samples reached a final moisture content of (0.077 ± 0.015) g water/g dry solid. After drying, the samples were vacuum-packed. All trials were performed in triplicate, and average values were used for analysis.	The study found that effective moisture diffusivity values for microwave-dried bee pollen ranged from 0.04 × 10⁻^8^ m^2^/s to 1.14 × 10⁻^8^ m^2^/s, with an activation energy of 71.68 W/g. Increasing microwave power levels from 180 w to 900 W significantly reduced drying time by 94%. Microwave power levels influenced various quality attributes of bee pollen: - Protein, fat, total carbohydrates, and vitamin C: All were affected by the microwave power level. - Color values: The color change (ΔE) was notably impacted, with the lowest ΔE value observed at the 180 W power level. - Solubility: The highest solubility was also noted in bee pollen dried at 180 W. Morphological changes were evident in microwave-dried bee pollen grains, but overall, bee pollen dried at 180 W power level retained its quality attributes better than those dried at higher power levels, considering physicochemical properties and organoleptic characteristics.	[62]
Changes in the structural and thermodynamic characteristics of bee pollen	Solar-drying	N/A	The study compared two drying methods for bee pollen: 1. Solar Dryer: - Temperature: Averaged 19–35 °C, peaking at 38 °C. - Relative humidity: Average of 55%. 2. Forced convection oven: - Temperature: Set at (55 ± 2) °C. - Relative humidity: Average of 10%. Analyses Conducted: - Phase transition enthalpy: Measured by DSC. - Porosity and surface area: Assessed through surface area analysis. - Microscopic surface appearance: Observed by SEM. The study aimed to evaluate the impact of these drying methods on various morphological and thermodynamic properties of bee pollen.	The study found the following results regarding the impact of drying methods on bee pollen: Morphological changes: No significant changes were observed in the surface appearance of dried bee pollen compared to fresh samples under SEM. Both cabin and solar-drying methods did not alter the morphological structure. Surface area and porosity: Surface area analysis indicated that neither solar nor cabin-drying affected the specific surface area or porosity of the bee pollen. The microscopic and macroscopic structure remained consistent with that of fresh bee pollen. Thermal analysis: DSC: Dried bee pollen samples showed endothermic phase transitions at 145 °C for cabin-dried pollen and 160 °C for solar-dried pollen. TGA: Confirmed the presence of phase transitions.	[61]
Changes in functional compounds profile	Sun-ray-drying (SD), shadow-drying (ShD), and incubation (ID)	N/A	The study involved drying pollen grains using different methods and temperatures: 1. Drying methods and temperatures: - Sun-ray-drying: 41 °C. - Shadow-drying: 32 °C. - Incubator-drying (ID): 32 °C, 40 °C, and 50 °C. 2. Drying time: - Samples were collected after 24 h, 48 h, and 72 h. 3. Storage: - All treated pollen grains were stored at −20 °C until analysis.	Phenolic compounds: Sun-ray-drying: Highest phenolic compound content after 72 h. Shadow-drying: Highest phenolic compound content after 72 h. Incubator drying: 32 °C: Highest number of phenolic compounds after 24 h. 40 °C: Highest number of phenolic compounds after 24 h. 50 °C: Highest number of phenolic compounds after 24 h.	[54]

## 8. Potential Applications of Bee Pollen Products in Nutrition and Medicine

Bee pollen has attracted significant attention within both the fields of nutrition and medicine due to its rich composition of bioactive compounds and its wide range of potential therapeutic uses [101]. Often hailed as a “complete food,” bee pollen contains essential nutrients like proteins, vitamins, minerals, amino acids, polyphenols, flavonoids, and essential fatty acids [102].

In nutrition, bee pollen has become a potent functional food. Its impressive nutritional profile, which includes vitamin A, C, E, and B complexes, along with minerals such as calcium, magnesium, iron, and zinc, supports several physiological functions and makes it an excellent dietary supplement [3]. Furthermore, its high concentration of amino acids, including essential ones like histidine, leucine, and lysine, aids in muscle repair, tissue growth, and overall metabolic health [103]. The presence of unsaturated fatty acids, such as omega-3 (ω-3), ω-6, and ω-9, contributes to its anti-inflammatory properties, promoting cardiovascular health and reducing the risk of chronic diseases like arthritis and cancer [104]. The antioxidant properties of bee pollen further elevate its importance in nutrition. Polyphenols and flavonoids, including quercetin and kaempferol, act as powerful antioxidants that neutralize free radicals, protecting cells from oxidative stress. This activity has been linked to the prevention of degenerative conditions, such as arteriosclerosis and cancer. Additionally, the carotenoids and vitamins found in bee pollen help reduce oxidative stress, a significant factor in aging and disease development [105].

Bee pollen’s technological properties make it a valuable ingredient in food processing [106]. Its capacity to retain oil and enhance flavor makes it ideal for products such as yogurts, candies, and juices. Not only can bee pollen improve texture and prolong shelf life, but it can also contribute to the overall mouthfeel of processed foods [3]. Research has shown that incorporating bee pollen into food products can increase the bioavailability of essential nutrients and antioxidants, enhancing the health benefits of those products [107].

Likewise, bee pollen’s medicinal properties have gained considerable attention, particularly due to the diversity of bioactive compounds that provide therapeutic effects. It has demonstrated antimicrobial, antifungal, antiviral, and anti-inflammatory activities, which contribute to the prevention and treatment of various conditions [4]. Its immunostimulant properties are believed to strengthen the immune system, potentially aiding in the prevention of infections and diseases [101].

Clinical studies have explored the role of bee pollen in managing chronic prostatitis and benign prostatic hyperplasia [101]. Pollen extracts have shown anti-inflammatory and anti-androgenic effects, alleviating symptoms and improving urinary flow in men suffering from these conditions. Research has found that flavonoids like quercetin, a key component of bee pollen, reduce inflammation and oxidative stress, which are crucial factors in prostate disorders. In cancer research, bee pollen has shown promising potential. Studies suggested that bee pollen can induce apoptosis, or programmed cell death, in cancer cells and inhibit tumor growth [101]. Some specific types of bee pollen, such as those derived from *Nelumbo nucifera* (lotus), have been found to inhibit the proliferation of prostate cancer cells [108]. Additionally, a combination of bee pollen and honey has been reported to alleviate menopausal symptoms in breast cancer patients undergoing antihormonal therapy, suggesting potential benefits in cancer care [109]. Bee pollen also holds promise in the treatment of allergies. When used in immunotherapy, bee pollen extracts have been shown to reduce the symptoms of allergic rhinitis, commonly known as hay fever. Immunotherapy with grass pollen extracts has been found to improve the quality of life for allergy sufferers by decreasing the severity of asthma and other allergic reactions [110].

In dermatology and cosmetics, bee pollen offers numerous benefits. Its rich phenolic and flavonoid content provides strong antioxidant and anti-melanogenic effects, helping to protect skin cells from UV damage and abnormal pigmentation [111]. Bee pollen’s ability to inhibit tyrosinase, an enzyme critical to melanin production, makes it a valuable ingredient in cosmetic products designed to treat age spots, freckles, and hyperpigmentation disorders [112]. Moreover, bee pollen has been incorporated into hair care products such as shampoos and conditioners due to its antifungal properties, particularly for treating dandruff [112]. In reference to the above, Table 2 lists some specific examples of pollen-based products and their main benefits.

## 9. Challenges in Commercializing Bee Pollen with Functional Properties

Currently, bee pollen is marketed as a dietary supplement or food ingredient due to the need for more standardization of processes and microbiological susceptibility. As a product, it is marketed in granule form, which has only undergone convection-drying processes [119]. Data on pollen consumption’s effects on living organisms’ functional properties are limited. This poses a challenge for the food industry, as the commercialization of products with nutritional and health claims must be supported by scientific studies validated by relevant health authorities.

In fermentation processes, bee bread could be processed as fermented pollen [9]. However, the natural production of bee bread is scarce, and the U.S. Food and Drug Administration (FDA) has not approved its sale due to the low aseptic conditions in which it is produced [120]. Some researchers suggest consuming bee bread in smaller amounts or for shorter durations than pollen [121]. On the other hand, studies have shown that pollen can increase its digestibility through grinding or soaking in warm water, increasing its polyphenol content by up to 11 times [122]. Other methods include soaking in milk, fruit, and vegetable juices [121]. For the commercial interests of the food industry, this represents a significant advantage, as these processes require less energy than other methods, such as drying or using bacteria and enzymes.

Some authors claim that unground pollen, when chewed carefully before swallowing, is utilized by the body at only 10%–15%. After mechanical grinding or natural release, the biological accessibility of pollen increases up to 60%–80% [8]. This should also be considered for pollen processing to obtain a final product with higher nutritional value, as the technical application might not be necessary given that the bioavailability of pollen improves with natural processes like chewing.

## 10. Conclusions

The great potential of bee pollen in nutrition and medicine is immense, while contributing to the sustainable development of communities with a focus on food security and sovereignty. Currently, some community associations in Colombia are already marketing pollen-based products such as snacks (https://mielatto.com/product-tag/apiario-el-pinar/; accessed on 24 October 2024). However, the exine of pollen could limit the bioavailability of its nutrients, so methods have been proposed to degrade the exine. The review of advanced methods such as fermentation, enzymatic hydrolysis, and ultrasonic treatment demonstrates their ability to transform bee pollen, improving its nutritional and bioactive properties by increasing the bioavailability of nutrients and phenolic compounds and degrading allergenic compounds. These methods also optimize digestibility, increase protein content, and promote higher antioxidant activity, making pollen a more valuable functional ingredient.

Pollen with a modified exine could have potential use in dietary supplements (*e.g.*, capsules, sprays, syrups, tablets, vials), functional foods (*e.g.*, snacks, blends with honey, candies), cosmetics (*e.g.*, creams, foaming products), and pharmaceuticals (*e.g.*, extracts) (*e.g.*, https://honeygreen.com/es/product/polen-de-abeja-farma/; accessed on 24 October 2024). The enhanced bioavailability makes bee pollen an ideal functional food, rich in amino acids, vitamins, and antioxidants that promote immune function, cardiovascular health, and skincare. In cosmetics, the anti-aging, antioxidant, and anti-inflammatory properties of pollen are valuable for skin and hair care products. In addition, in pharmaceuticals, pollen extracts can help treat conditions such as prostate disorders, allergies, and inflammatory diseases. However, the practical application of the reviewed techniques requires a thorough understanding of the biochemical mechanisms involved and continuous process optimization to ensure that the final product meets the quality and safety standards of the food industry. Further studies are needed to demonstrate the need for pollen exine modification. Some other challenges include the standardization of processing techniques, commercial scalability of methods, optimizing exine degradation to preserve nutrient integrity, and addressing allergenic potential. In addition, clinical trials are needed to validate the health benefits of exine-treated pollen, and regulatory hurdles for market approval must be overcome.

As research progresses, it is essential to establish standardized protocols and clear guidelines for producing processed bee pollen, thus enabling its effective integration into the functional food industry and ensuring that consumers can fully benefit from its enhanced properties. So, future research should focus on developing more cost-effective and scalable exine treatments, improving nutrient bioavailability, and conducting clinical studies on their effects. In addition, advanced technologies such as biotechnology and nanotechnology could improve the extraction and distribution of pollen in medical devices. Addressing these challenges will help harness the full potential of bee pollen in global health markets.

Finally, the literature reports that, for specific treatment purposes, the transformation technique applied will influence the final product. Therefore, it is necessary to increase the number of studies confirming the need to apply methods for modifying pollen’s exine. This would enable a greater market availability of pollen-based products with functional properties.

## Figures and Tables

**Table 2:** Examples of commercial pollen-based products.

Product	Country	Field	Form	Ingredients	Primary Uses
Bee pollen capsules [113]	United States	Medicine	Capsules	Organic bee pollen, vitamin C powder, organic protein powder	High-protein dietary supplement, anti-fatigue, immune support, enhances circulation, allergy relief
Cosmetic applications [114]	Various	Medicine	Creams, lotions	Bee pollen (flavonoids, polyphenols)	Protects skin cells from abnormal melanogenesis, reduces age spots, freckles, melasma, malignant melanoma
Bee pollen tonics [115]	Various	Medicine	Liquid tonics	Bee pollen, herbal extracts, vitamins	General tonic for immune boosting, fatigue reduction, and overall health
Propovitamin adultos [116]	Colombia	Nutrition	Oral liquid	Honey, propolis, bee pollen, vitamin A, vitamin C, glucose liquid, antioxidants	Nutritional supplement supporting immune health, rich in vitamins and propolis benefits
AlivMiel candy [117]	Colombia	Nutrition	Candy	Sugar, honey, glucose, peppermint essence, lemon/cherry essence, propolis, bee pollen, vitamin C, artificial flavorings	Candy rich in propolis and pollen for immune support, vitamin C boost, sore throat relief
Bee pollen granules [118]	Various	Nutrition	Granules	Bee pollen	Nutritional supplements in raw form, high in essential amino acids, vitamins, and minerals

## Data Availability

No new data were created or analyzed in this study. Data sharing is not applicable to this article.
